# Effects of preoperative oral carbohydrate intake on catabolism, nutrition and adipocytokines during minor surgery: A randomized, prospective, controlled clinical phase II trial

**DOI:** 10.1371/journal.pone.0216525

**Published:** 2019-05-13

**Authors:** Yoshinari Morimoto, Tomoko Kinugawa, Megumi Hayashi, Takatoshi Iida, Tatsuo Yamamoto

**Affiliations:** 1 Department of Critical Care Medicine and Dentistry, Graduate School of Dentistry, Kanagawa Dental University, Yokosuka, Kanagawa, Japan; 2 Department of Oral Science, Graduate School of Dentistry, Kanagawa Dental University, Yokosuka, Kanagawa, Japan; Universita degli Studi di Milano-Bicocca, ITALY

## Abstract

**Background:**

We investigated the effects of preoperative oral carbohydrate loading on intraoperative catabolism, nutritional parameters, and adipocytokine levels during anesthesia.

**Methods:**

Study participants were randomized to two groups who were allowed to consume either no more than 250 mL of 18% oral carbohydrate solution (Arginaid Water: AW group) or no more than 500 mL of plain water (PW group) within the 2 hours before surgery, with no intraoperative glucose administration. Percentage changes from preoperative values of resting metabolic rate (RMR) and total body water (TBW), determined by bioelectrical impedance analysis (BIA), were compared. Blood levels of serum ketone bodies, free fatty acids (FFAs), insulin, 3-methyl histidine, blood glucose, retinol binding protein, adiponectin, and leptin were measured. BIA measurement and blood sampling were performed on entry to the operating room (M1) and 2 hours after the induction of anesthesia (M2). Chi squared test, Mann-Whitney U test, and Wilcoxon’s test were used for comparisons of parameters. *P* values less than 0.05 constituted a significant difference.

**Results:**

Seventeen patients per group (34 patients total) were enrolled. RMR and TBW values did not differ between M1 and M2 measurements. Participants in the AW group had lower blood ketone body and FFA levels and higher insulin levels at M1. However, their ketone body and FFA levels rose and insulin levels fell after 2 hours, although ketone body and FFA levels in the AW group were still lower than those in the PW group. Although retinol binding protein, adiponectin, and leptin levels were not different in terms of preoperative oral carbohydrate loading, the levels of these substances in both groups were lower after 2 hours compared with levels on operating room entry.

**Conclusions:**

Preoperative oral carbohydrate loading without intraoperative glucose administration appears to suppress catabolism for 2 hours after the start of surgery.

## Introduction

Preoperative fasting results in mobilization of lipids from stored glycogen in the liver [1,2], since glycogen stores are below the amount required for one day’s basal metabolism, and from increased catabolism of muscle protein, which results in ketone body elevations [3,4]. The resulting increase in insulin resistance, moreover, requires eight times the normal amount of insulin volume to maintain postoperative blood glucose at normal levels [4].

Surgical patients on an enhanced recovery after surgery (ERAS) program are given 800 mL of a 12.6% oral carbohydrate solution by the middle of the night before surgery, with an additional 400 mL of the solution by 2 hours before surgery. This has the effects of increasing insulin secretion, maintaining glycogen stores, inhibiting protein breakdown, suppressing the secretion of catabolic hormones (cortisol, catecholamines), and suppressing catabolism [5–9]. Systematic reviews and meta-analysis of ERAS studies have shown decreased postoperative insulin resistance with preoperative carbohydrate loading, but have not clearly demonstrated the efficacy in terms of shorter hospital stays, lower postoperative infection rates, maintenance of muscle mass and strength, and lower rates of postoperative nausea and vomiting [10–12].

The stress response of the endocrine and metabolic systems to the insult of surgery lowers insulin secretion, which in turn lowers insulin sensitivity, increases the release of catabolic hormones, reduces glucose utilization, and increases gluconeogenesis, thus increasing blood glucose levels [13–16]. Many studies have found that administration of low-dose glucose solutions during surgery, on the other hand, suppresses the level of blood ketone bodies and free fatty acids (FFAs) without substantially increasing blood glucose levels, and also increases insulin levels [3,4,13,14,17].

Intraoperative catabolism is also affected by the invasiveness of the surgery, the type of anesthesia, blood loss and body temperature, although no studies have evaluated lipid and protein catabolism with these intraoperative parameters held constant. One method of maintaining these intraoperative factors constant would be to perform metabolic investigations during minor surgeries that have constant invasiveness and minimal blood loss, and that were performed under a single anesthesia method. Furthermore, in the ERAS studies, because all parameters were measured up to the point of anesthesia induction, it is unclear if the suppression of catabolism is due to the effect of preoperative oral carbohydrate administration or due to intraoperative infusion of solutions with/without glucose [5,6].

The ERAS program recommends a total of 1200 mL of preoperative fluids. However, Asians, due to their smaller body size compared with Westerners, are likely to be unable to consume this entire volume. In Japan, due to the unavailability of 12.6% oral carbohydrate solutions, an 18% oral carbohydrate solution (Arginaid Water; Nestle Health Science Co., Tokyo, Japan)] is used for preoperative fluid intake [18,19]. The manual for Arginaid Water recommends consumption of 1 pack (125 mL) daily [20]. However, no study has evaluated lipid and protein catabolism and nutritional parameters during surgery in patients given such a realistically consumable amount of fluid. Further, adipocytokines have recently received focus as factors relevant to increases in insulin sensitivity, although it is unknown how the levels of these factors change during surgery in response to preoperative oral carbohydrate loading.

The objective of the present phase II trial was to compare changes in intraoperative lipid and protein catabolism, nutritional parameters and adipocytokine levels in a group of participants preoperatively receiving an oral carbohydrate solution and a group given water alone, to evaluate the effects and limitations of preoperative oral carbohydrate loading in an environment isolated from other factors. To strictly evaluate the efficacy of preoperative intake of oral carbohydrate solution, this study was designed to maintain surgical invasiveness, blood loss and body temperature at constant levels by achieving proper anesthetic depth and body fluid levels, and to perform minor surgery without glucose administration in order to eliminate the effects of these variables on blood glucose and metabolism during surgery.

## Materials and methods

### Study design and patient population

This study was conducted according to the principles of the Declaration of Helsinki. The protocol was approved by the Institutional Research Board and Ethics Committee of Kanagawa Dental University (approval No. 390). The participants were those who gave written informed consent after being thoroughly informed of the study methods, benefits, safety and risks. The protocol was registered and published in the University Hospital Medical Information Network Center (UMIN Clinical Trials Registry) (ID: UMIN000021958, registered on May 1, 2016).

**Fig 1:**
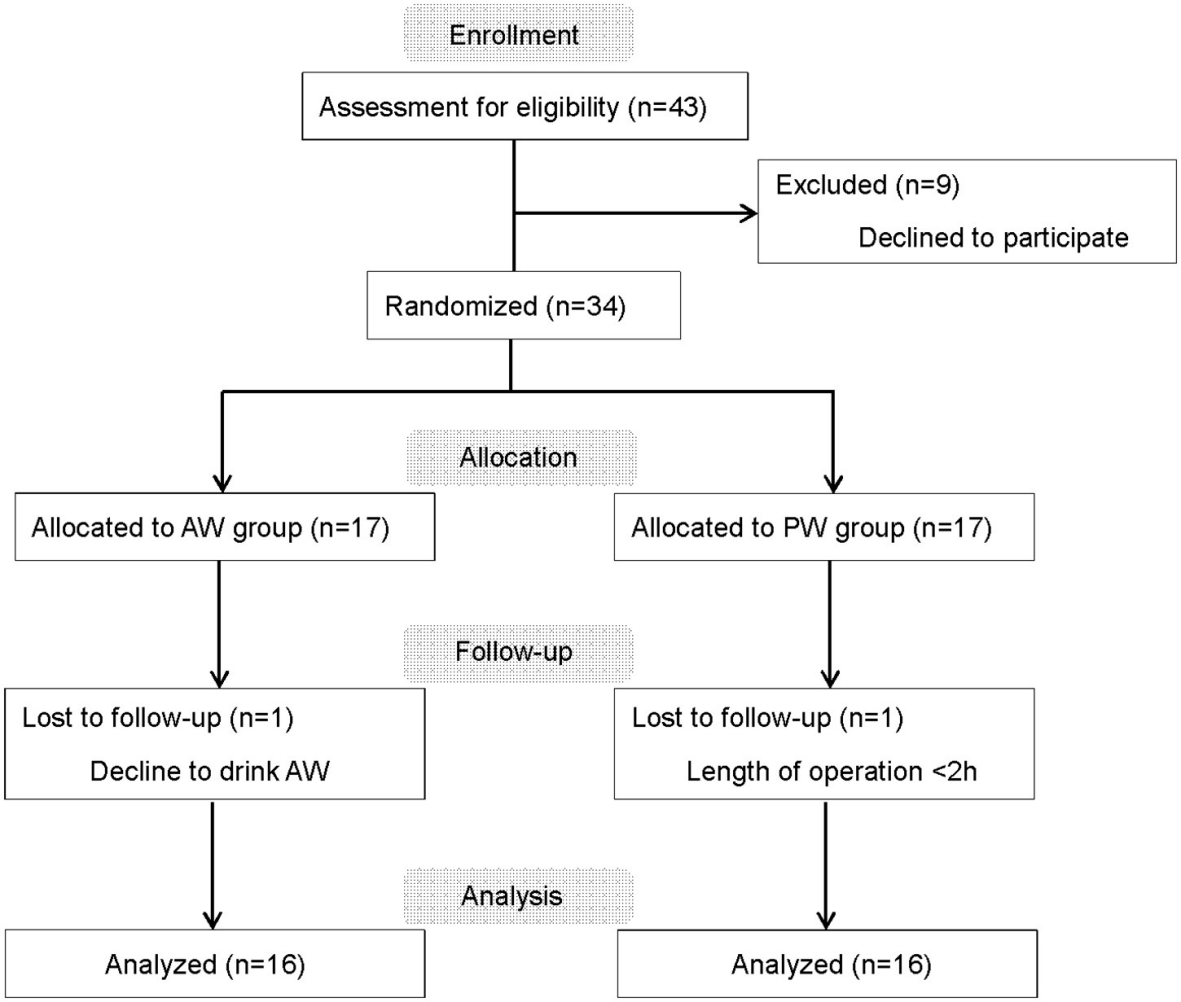
CONSORT flow chart showing the selection process for patients. AW: Arginaid water, PW: plain water.

The participants were patients aged 18 to 65 years, with an American Society of Anesthesiologists (ASA) physical status (PS) score of 1 or 2, who were scheduled for oral surgery under general anesthesia at the Kanagawa Dental University Hospital from July 2016 to August 2017. The studied procedures were surgeries with a scheduled operating room entry time of between 9:10 a.m. to 9:30 a.m., and an estimated duration of anesthesia of at least 2 hours and blood loss of no more than 50 mL during the first 2 hours of anesthesia. The exclusion criteria were patients with diabetes mellitus, thyroid disease, or other metabolic abnormalities, patients on steroid hormone products or α or β receptor agonists with metabolic effects, patients who were obese (i.e., BMI >28 kg/m^2^) or abnormally underweight (i.e., BMI <17 kg/m^2^), patients unable to consume normal meals because of a digestive disorder or psychiatric disease (anorexia nervosa), and patients unable to undergo body composition measurement by bioelectrical impedance analysis (BIA) due to a limb disease. All patients who met the eligibility criteria described above during the study period were informed of the study methods, and 34 of those who gave informed consent were included in this study. The participants were randomly assigned to one of two groups, the Arginaid Water (AW) group and the plain water (PW) group, using computer-generated random numbers (simple randomization by chance was employed). Seventeen subjects were included in each group. One participant in the AW group was excluded after refusing to consume Arginaid Water preoperatively. One participant in the PW group was excluded because the duration of anesthesia was less than 2 hours. Ultimately, the AW and PW groups included 16 participants each (Fig 1). Someone (MH, TI) other than the anesthesiologist independently controlled the group assignments, type and amount of fluid, and the time of the last drink. This information was not shared with the anesthesiologists (YM, TK).

### Preoperative body composition measurement

The participants were admitted at 10:00 a.m. on the day before surgery, and ate lunch at 12:00 p.m. and supper at 6:00 p.m. To exclude the effects of increased meal-related metabolism and body fluid levels on body composition measurements, the participants underwent BIA [BioScan 920-II (Maltron International Ltd., England)] between 4:00 and 6:00 p.m. after resting in bed for 20 minutes in a quiet room at a temperature of approximately 25°C, to measure their resting metabolic rate (RMR) and total body water content (TBW) as baseline values to measure metabolic status and body water volume. Anesthesiologists were not informed of the results of preoperative measurements.

### Principles of BIA

BIA measures the resistance (impedance) to passage of a weak, harmless electrical current through the body [21]. The water content of muscle is a good conductor of electricity. People with higher amounts of muscle, therefore, have lower impedance, while those with higher amounts of fat have higher impedance. Changes in electrical resistance occurring when body fluid levels fluctuate, however, result in spurious changes in measured body fat levels, even though the actual level of body fat remains the same, meaning that extra fluid levels in a person who has received intravenous fluids may be mistaken for extra skeletal muscle [22,23]. Measurements such as body muscle and fat percentage that are affected by body fluid were not compared in this study.

### Preoperative fluid intake

The participants were not allowed to eat or drink from 9:00 p.m. on the day before surgery until entry into the operating room, but were allowed to consume either no more than 250 mL of AW or no more than 500 mL of PW from 9:00 p.m. on the day before surgery to 7:00 a.m. on the day of surgery, based on their randomization. AW contains carbohydrates: dextrin and sucrose, 18 g/dL; phosphate, 180 mg/dL; zinc, 8 mg/dL; copper, 0.8 mg/dL; and arginine, 2.0 g/dL (osmotic pressure, 560–580 mOsm/L; 80 kcal/dL). The manual for AW recommends consumption of no more than 1 pack (125 mL) a day, so the participants were given 2 packs (250 mL) to consume, one on the day before and the other on the day of surgery [20]. At 7:30 a.m. on the day of surgery, the AW and PW containers were collected, and the volume remaining was measured in a graduated cylinder to determine the volume consumed. The participants were asked to state the time they took their last drink.

### Anesthesia

No premedication was administered. The patients were brought into the operating room between 9:10 and 9:30 a.m. On entry, a non-invasive sphygmomanometer, electrocardiograph and pulse oximeter were attached. Then, the electrodes of the BioScan 920-II were attached and the first body composition measurement was taken. With peripheral vein access gained via a 20 G indwelling needle inserted into the back of the left or right hand, the first blood sample (M1) was collected, following which 6 mL/kg/hour of acetated Ringer’s solution (Solacet F: Terumo Co. Tokyo, Japan) was delivered. General anesthesia was induced with an intravenous infusion of 100 μg of fentanyl followed by boluses of 2 mg/kg of propofol and 0.6 mg/kg of rocuronium bromide, and maintained with an infusion of remifentanil 0.3 μg/kg/min. Mask ventilation with 6 L of oxygen per minute and 4% to 5% sevoflurane was given for 3 minutes. Nasotracheal intubation was performed after the end-tidal sevoflurane concentration (etSev) was confirmed to be at least 3.0%. Mask ventilation was continued if etSev had not yet reached 3.0% at this point, with intubation performed when etSev reached at least 3.0%. After intubation, anesthesia was maintained with 1 L/minute oxygen, 2 L/minute air, 2% sevoflurane, and 0.1 to 0.5 μg/kg/min of remifentanil. Remifentanil was adjusted to keep blood pressure and pulse rate within ± 25% of the values just before anesthesia induction. Ventilator settings were adjusted to achieve a tidal volume of 6 to 9 mL/kg per breath and 10 to 14 respirations per minute. Respiration was controlled to achieve an end-tidal CO_2_ concentration of 30 to 35 mm Hg. The second body composition measurement and blood sampling (M2) were performed 2 hours after the induction of anesthesia. Participants were warmed from their entry into the operating room. A rectal temperature of 36–37°C was maintained throughout the surgery.

All participants were trans-nasally intubated. After anesthesia was induced, left and right nostril patency was determined using a cotton swab during mask ventilation. About 1.0 cc of 4% lidocaine hydrochloride spray was sprayed into the more patent nostril to achieve topical anesthesia. An Ivory PVC, Nasal, Soft Seal Cuff Tracheal Tube (Smith Medical Japan Ltd., Tokyo) (inner diameter of 7.0 mm for men and 6.5 mm for women) was inserted into the selected nostril. Only anesthesiologists with at least 1 year of experience were involved in the study.

### Measurements

Sex, age, and BMI were determined from the medical records of the participants. Other preoperative and intraoperative information evaluated included the type of fluid (AW or PW), amount consumed, time since consumption (time from last drink to entry into the operating room), blood loss and urine output up to 2 hours after the start of anesthesia, and mean remifentanil dose used (μg/kg/min).

#### Blood tests

Blood tests were conducted to determine serum total ketone bodies, β-hydroxybutyrate, acetoacetate, free fatty acids (FFAs), insulin, 3-methyl histidine (3-MH), blood glucose, retinol binding protein, adiponectin and leptin levels. Blood samples were centrifuged at our hospital laboratory, and the resultant serum was cryopreserved at -20°C and analyzed by the testing facility (LSI Medience Corporation, Tokyo, Japan) on the day of collection.

#### BIA measurements

The BIA measurements of resting metabolic rate (RMR) and total body water (TBW) were compared. The percentage changes in pre-anesthesia (M1) and 2-hour (M2) measurements were assessed relative to the previous day’s measurements (preoperative values), because baseline measurements varied among the participants.

### Amount and time of fluid consumption

The correlation between the amount and timing (time from last drink to entry into the operating room) of preoperative fluid consumption, and total ketone bodies at M1 and M2 were determined in the AW and PW groups.

### Statistical analysis

Statistical analysis was performed with SPSS version 16.0 software (SPSS Japan, Tokyo, Japan). Data are expressed as medians (and quartiles). A chi squared test was used to analyze male-to-female ratios. The Mann-Whitney U test was used to compare other parameters between the AW and PW groups. Wilcoxon’s signed-rank test was used for intra-group comparisons of M1 and M2 values. Spearman’s rank correlation coefficient was used to analyze the correlation between the amount and time of preoperative fluid consumption and total ketone bodies in M1 and M2. In all cases, *P* values less than 0.05 constituted a significant difference.

Total ketone bodies in the blood was the primary endpoint. When determining the required number of patients, we found that a sample size of 14 patients per group would provide a power of 80% at an alpha error of 0.05 and beta error of 0.20 using a two-tailed t-test, based on the findings of a comparison of total blood ketone bodies in patients intraoperatively receiving intravenous fluids with and without 1% glucose, in which the blood glucose level at the end of surgery was about 200 ± 180 mmol/L (mean ± standard deviation) in patients receiving fluids with 1% glucose and about 450 ± 260 mmol/L in patients given fluids without glucose [13]. The average standard deviation (220) was employed for calculating the required number of cases. We ultimately determined that 17 patients per group (34 patients in all) would be necessary, assuming a 20% dropout rate. In these procedures, we initially tried to calculate the sample size based on preoperative intake of an 18% oral carbohydrate solution [18]. However, since the obtained sample size was 12 patients per group because of the large difference in outcomes between groups, we were concerned that the smaller sample size might have had misleading results. Therefore, the sample size was eventually calculated based on the report of intraoperative glucose infusion in a similar category [13].

The original study protocol submitted to the Institutional Research Board and Ethics Committee of Kanagawa Dental University included infants and children as participants. However, as surgeries for such patients were rare in our hospital, these patients were not enrolled in this study.

## Results

### Patient baseline characteristics

The 32 participants (18 men and 14 women) had a median age of 35 years (range of 19 to 65 years), median BMI of 22.1 (range of 17.5 to 26.6), median blood loss within 2 hours after the start of anesthesia of 20 mL (quartiles were 5 to 30 mL), and median urine output of 60 mL (quartiles were 25 to 150 mL). The maximum blood loss was 62 mL. The surgical procedures included 11 tooth extractions, 11 removals of a metal plate after surgery for jaw deformity, three jaw cyst surgeries, two plastic surgeries for mandibular torus, two surgeries to remove tongue leukoplakia, two surgeries to remove benign tumors, and one sialolithotomy (Table 1). No adverse events were apparent clinically throughout this study.

**Table 1:** Types of surgical procedures performed.

	Arginaid Water	Plain Water
Tooth extraction	4	7
Metal plate removal after surgery for jaw deformity	5	6
Jaw cyst surgery	2	1
Plastic surgeries for mandibular torus	1	1
Removal of tongue leukoplakia	2	0
Removal of benign tumors	1	1
Sialolithotomy	1	0

### Comparison of patient characteristics and body composition

No differences between the AW and PW groups were noted in age, sex, BMI, amount of fluid consumed, blood loss and urine output up to 2 hours after the start of surgery, or amount of remifentanil used. The time from the last drink to operating room entry, however, was significantly shorter in the PW group (Table 2).

**Table 2:** Patient characteristics, changes in body composition and relationship between preoperative intake and ketone body values.

		Arginaid Water	Plain Water	P value
Age (years)		40.5 (28.25–61.0)	31.5 (24.75–56.0)	0.491
Sex (male/female)		9/7	9/7	1
BMI		22.75 (21.35–24.48)	20.95 (20.20–23.28)	0.094
Volume of fluid consumed preoperatively (mL)		250 (250–250)	255.25 (178.75–500)	0.564
Time interval of intake before anesthesia (min)		365 (210–615)	215 (181.25–300)	0.026
Blood loss (mL)		20 (5–37.5)	15 (5–35)	0.669
Urine volume (mL)		122.5 (52.5–200)	55.0 (10–150)	0.184
Remifentanil used (μg/kg/min)		0.098 (0.075–0.136)	0.102 (0.075–0.135)	0.926
Changes in BIA values				
Resting metabolic rate (%)	M1	-0.45 (-1.19–0.415)^a1^	-0.38 (-0.78–0.44)^a2^	0.809
	M2	-0.20 (-0.76–0.65)^a1^	-0.08 (-1.20–1.30)^a2^	0.724
Total body water (%)	M1	-2.45 (-4.33–0.93)^b1^	-1.19 (-3.79–1.80)^b2^	0.724
	M2	-1.06 (-4.05–2.13)^b1^	0.03 (-3.31–4.32)^b2^	0.423

Abbreviations: M1: measurement of body composition at the start of anesthesia

M2: measurement of body composition 2 h after the start of anesthesia

BIA: bioelectrical impedance analysis

Data are presented as median (25–75% quartile). Statistical differences between M1 and M2 were as follows:

^a1^ P = 0.550,

^a2^ P = 0.179,

^b1^ P = 0.346, and

^b2^ P = 0.088.

Body composition measurements revealed no differences between the AW and PW groups in RMR and TBW at both M1 and M2. Intragroup comparisons of M1 and M2 values in the AW and PW groups, likewise, revealed no differences (Table 2).

### Comparison of biochemical parameters between AW and PW groups

**Fig 2:**
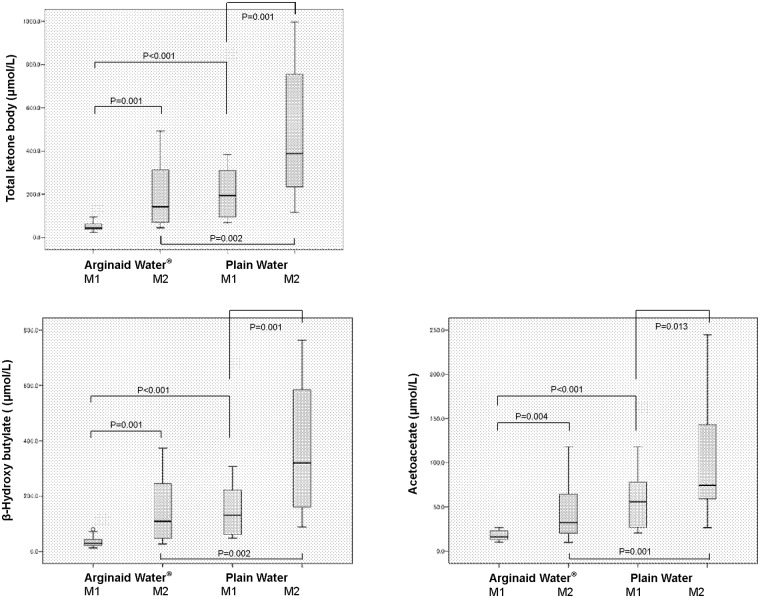
Serum concentration of ketone bodies in surgical patients. A: Total ketone body levels were 43.75 (38.75–64.25) μmol/L and 141.5 (64.18–340.50) μmol/L at M1 and M2 in the carbohydrate (AW) group, and 193.50 (93.05–331.25) μmol/L and 388.50 (229.50–815.75) μmol/L at M1 and M2 in the plain water (PW) group. B: β-hydroxybutyrate levels were 29.55 (22.30–44.33) μmol/L and 109.40 (42.90–266.75) μmol/L at M1 and M2 in the carbohydrate (AW) group, and 131.00 (60.43–231.00) μmol/L and 320.00 (154.75–632.00) μmol/L at M1 and M2 in the plain water (PW) group. C: Acetoacetate levels were 16.15 (13.05–23.28) μmol/L and 32.35 (20.28–67.10) μmol/L at M1 and M2 in the carbohydrate (AW) group, and 55.85 (26.60–78.10) μmol/L and 74.55 (55.53–144.00) μmol/L at M1 and M2 in the plain water (PW) group.

**Fig 3:**
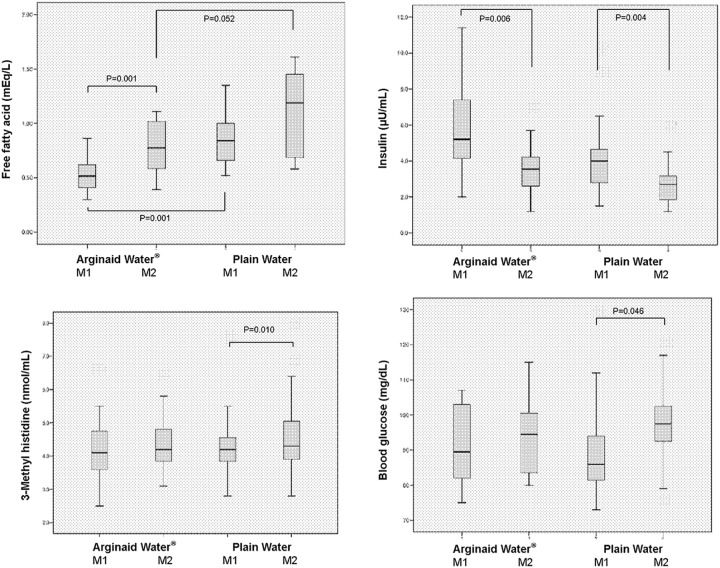
Serum concentration of free fatty acids, insulin, 3-methyl histidine and blood glucose in dental surgical patients. A: Free fatty acid levels were 0.515 (0.405–0.625) mEq/L and 0.775 (0.583–1.030) mEq/L at M1 and M2 in the carbohydrate (AW) group and 0.840 (0.640–1.175) mEq/L at M1 and 1.190 (0.643–1.530) mEq/L at M1 and M2 in the plain water (PW) group. B: Insulin levels were 5.20 (4.13–8.10) μU/mL and 3.55 (2.45–4.30) μU/mL at M1 and M2 in the carbohydrate (AW) group and 4.00 (2.80–4.83) μU/mL and 2.70 (1.80–3.20) μU/mL at M1 and M2 in the plain water (PW) group. C: 3-methyl histidine (3-MH) levels were 4.10 (3.55–4.78) nmol/mL and 4.20 (3.83–4.80) nmol/mL at M1 and M2 in the carbohydrate (AW) group, and 4.20 (3.83–5.28) nmol/mL and 4.30 (3.80–6.40) nmol/mL at M1 and M2 in the plain water (PW) group. D: Blood glucose levels were 89.5 (81.0–103.0) mg/dL and 94.5 (83.3–101.3) mg/dL at M1 and M2 in the carbohydrate (AW) group, and 86.0 (81.3–94.5) mg/dL and 97.5 (92.3–103.3) mg/dL at M1 and M2 in the plain water (PW) group.

Total ketone bodies [M1: 43.75 (38.75–64.25) μmol/L and 193.50 (93.05–331.25) μmol/L, P<0.001; M2: 141.5 (64.18–340.50) μmol/L and 388.50 (229.50–815.75) μmol/L, in AW and PW groups, respectively, P = 0.002], β-hydroxybutyrate [M1: 29.55 (22.30–44.33) μmol/L and 131.00 (60.43–231.00) μmol/L, P<0.001; M2: 109.40 (42.90–266.75) μmol/L and 320.00 (154.75–632.00) μmol/L, in AW and PW groups, respectively, P = 0.002], and acetoacetate [M1: 16.15 (13.05–23.28) μmol/L and 55.85 (26.60–78.10) μmol/L, P<0.001; M2: 32.35 (20.28–67.10) μmol/L and 74.55 (55.53–144.00) μmol/L, in AW and PW groups, respectively, P = 0.001] were significantly lower in the AW group at both M1 and M2. FFA was lower in the AW group at both M1 and M2 [M1: 0.515 (0.405–0.625) mEq/L and 0.840 (0.640–1.175) mEq/L, P = 0.001; M2: 0.775 (0.583–1.030) mEq/L and 1.190 (0.643–1.530) mEq/L, in AW and PW groups, respectively, P = 0.052]. The values of the other parameters did not differ between the two groups (Figs 2 and 3). The amount and timing of preoperative fluid consumption did not correlate with total ketone bodies at M1 and M2 in both the AW and PW groups (Table 3).

**Table 3:** Relationship between preoperative intake and ketone body levels.

	Arginaid Water	Plain Water
M1 volume	r = -0.116 (P = 0.667)	r = 0.136 (P = 0.616)
time	r = -0.233 (P = 0.407)	r = -0.201 (P = 0.455)
M2 volume	r = 0.367 (P = 0.162)	r = 0.081 (P = 0.767)
time	r = 0.295 (P = 0.267)	r = -0.053 (P = 0.845)

### Intra-group comparisons

In the AW group, total ketone bodies (P = 0.001), β-hydroxybutyrate (P = 0.001), acetoacetate (P = 0.004) and FFA (P = 0.001) levels were higher at M2 than at M1, and insulin was lower at M2 than at M1 [5.20 (4.13–8.10) μU/mL at M1 and 3.55 (2.45–4.30) μU/mL at M2; P = 0.006]. Neither 3-MH nor blood glucose levels differed between M1 and M2 in the AW group (Figs 2 and 3).

In the PW group as well, total ketone bodies (P = 0.001), β-hydroxybutyrate (P = 0.001), acetoacetate (P = 0.013), 3-MH [4.20 (3.83–5.28) nmol/mL at M1 and 4.30 (3.80–6.40) nmol/mL at M2; P = 0.010) and blood glucose [86.0 (81.3–94.5) mg/dL at M1 and 97.5 (92.3–103.3) mg/dL at M2; P = 0.046) were higher at M2 than at M1, and insulin was lower at M2 than M1 [4.00 (2.80–4.83) μU/mL at M1 and 2.70 (1.80–3.20) μU/mL at M2; P = 0.004]. FFA did not differ between M1 and M2 in the PW group (Figs 2 and 3).

### Comparison of nutritional parameters and adipocytokine levels

**Fig 4:**
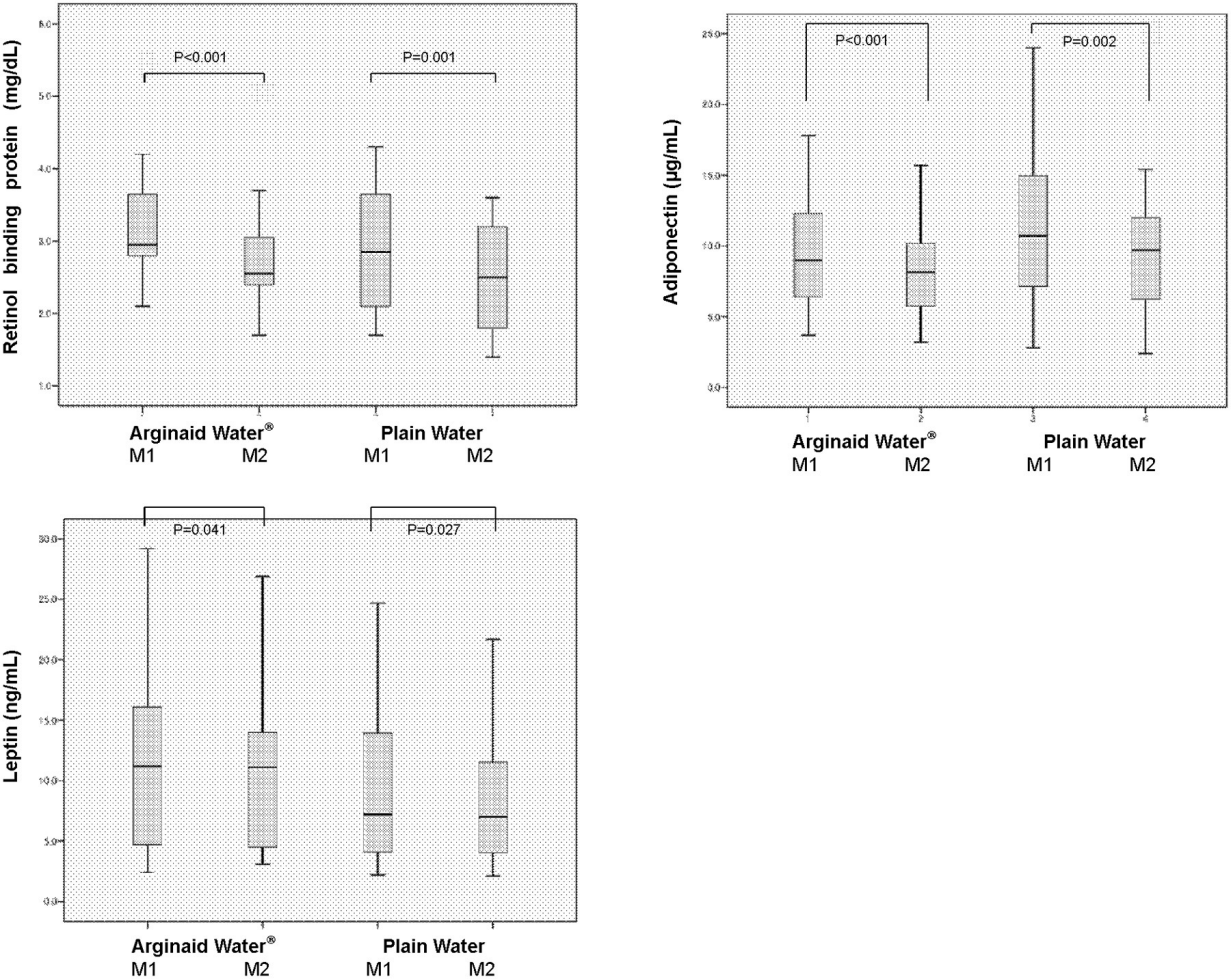
Serum concentration of retinol binding protein, adiponectin and leptin in dental surgical patients. A: Retinol binding protein levels: 2.95 (2.80–3.68) mg/dL and 2.55 (2.40–3.13) mg/dL at M1 and M2 in the carbohydrate (AW) group; 2.85 (2.05–3.73) mg/dL and 2.50 (1.70–3.40) mg/dL at M1 and M2 in the plain water (PW) group. B: Adiponectin levels: 9.00 (6.35–12.35) μg/mL and 8.15 (5.68–10.45) μg/mL at M1 and M2 in the carbohydrate (AW) group; 10.70 (7.08–15.23) μg/mL and 9.70 (6.00–12.40) μg/mL at M1 and M2 in the plain water (PW) group. C: Leptin levels: 11.20 (4.60–17.25) ng/mL and 11.10 (4.30–14.30) ng/mL at M1 and M2 in the carbohydrate (AW) group; 7.20 (4.10–14.03) ng/mL and 7.00 (4.00–13.30) ng/mL at M1 and M2 in the plain water (PW) group.

Retinol binding protein, adiponectin and leptin levels at M1 and M2 did not differ between the AW and PW groups. Further, while these parameters were lower at M2 than at M1 in both the AW and PW groups, all values were within the normal ranges [retinol binding protein: 2.95 (2.80–3.68) mg/dL and 2.55 (2.40–3.13) mg/dL at M1 and M2 in the AW group, P<0.001, and 2.85 (2.05–3.73) mg/dL and 2.50 (1.70–3.40) mg/dL at M1 and M2 in the PW group, P = 0.001; adiponectin: 9.00 (6.35–12.35) μg/mL and 8.15 (5.68–10.45) μg/mL at M1 and M2 in the AW group, P<0.001, and 10.70 (7.08–15.23) μg/mL and 9.70 (6.00–12.40) μg/mL at M1 and M2 in the PW group, P = 0.002; leptin: 11.20 (4.60–17.25) ng/mL and 11.10 (4.30–14.30) ng/mL at M1 and M2 in the AW group, P = 0.041, and 7.20 (4.10–14.03) ng/mL and 7.00 (4.00–13.30) ng/mL at M1 and M2 in the PW group, P = 0.027] (Fig 4).

## Discussion

In this study, we found that participants receiving preoperative oral carbohydrate loading had lower blood ketone body and FFA levels and higher insulin levels on entry to the operating room. However, in all the participants, ketone body and FFA levels rose and insulin levels fell after 2 hours, probably due to the fact that none of them received glucose intraoperatively. However, ketone body and FFA levels were still lower in the AW group than in the PW group 2 hours after the start of anesthesia. Although retinol binding protein, adiponectin and leptin levels did not differ according to whether or not oral carbohydrate loading was given preoperatively, the levels of these substances in both groups were lower after 2 hours compared with the levels at operating room entry.

Glucose metabolism and blood glucose levels during surgery depend on the invasiveness of the surgery, the types of anesthesia and anesthetics used, and body temperature, among other factors. Patients undergoing surgery under general anesthesia with a volatile anesthetic (halothane) have higher blood glucose and plasma cortisol levels than do patients receiving epidural block [24,25]. Unlike anesthesia with propofol and an opioid analgesic (sufentanil, fentanyl), anesthesia with a volatile gas (enflurane, isoflurane, sevoflurane) cannot sufficiently block the invasiveness of surgery, which leads to elevated plasma cortisol levels and a failure to sufficiently suppress the endocrine stress response [26–29]. Administering glucose to a patient under these conditions suppresses lipid and protein catabolism, but tends to cause exaggerated hyperglycemia [3,4,15,16]. Anesthesia with fentanyl or remifentanil, on the other hand, suppresses the stress response and reduces ACTH and cortisol levels regardless of the extent of surgery, and, thus, inhibits lipid mobilization without triggering hyperglycemia following low-dose glucose administration [17,19].

We controlled levels of oxygen, sevoflurane, fentanyl and remifentanil during anesthesia in this study, in reference to the methods of Tsutsumi and colleagues [19] and Sawada and colleagues [17], and also kept the degree of surgical invasiveness low. Intraoperative RMR, determined using BIA, remained stable, decreasing only slightly below the preoperative value, and blood loss was also low. TBW was, therefore, comparable to preoperative levels, and body temperature was well controlled. These parameters, thus, minimally affected metabolism. Since we did not administer glucose during surgery, the lipid and protein catabolism effects measured in the study were solely dependent on whether or not preoperative oral carbohydrate loading was given. Although the PW group was allowed to consume more fluids preoperatively than the AW group, this did not impact the results because actual consumption did not differ between the groups.

As previously reported, intraoperative low-dose glucose administration in fasting rats inhibited the catabolism of lipids and muscle protein [30,31]. Solutions of 1% to 1.5% glucose administered in clinical studies suppressed elevations in blood ketone bodies, FFA, and the amino acid 3-MH from skeletal muscle protein, increased insulin, and lowered postoperative insulin resistance [3,4,13,14]. Blood glucose remained below 150 mg/dL in all these studies [3,4,13,14]. Intraoperative administration of 2 mg/kg/min of glucose during major surgeries lasting at least 6 hours inhibited elevation of blood ketone bodies and the urinary 3-MH/creatinine ratio and maintained insulin levels [17].

In studies on the effects of oral carbohydrate loading with an 18% carbohydrate beverage (Arginaid Water) on protein catabolism, a dose of 650 mL of AW reduced insulin resistance in subjects who did not undergo surgery, and a dose of 500 mL suppressed pre-anesthesia blood ketone body and FFA levels [18,19]. These findings are similar to those of our study, in that the patients who received preoperative oral carbohydrate loading had lower pre-anesthesia ketone body and FFA levels and higher insulin levels compared with those who received water alone. Measurements taken after 2 hours without intraoperative glucose administration, moreover, revealed a smaller increase in ketone bodies in the preoperative oral carbohydrate loading group compared with the water-only group. Ketone bodies and FFA were significantly lower, and the increase in 3-MH was also inhibited. However, the fact that ketone bodies and FFAs increased and insulin decreased after measurement at 2 hours in both groups shows that, in the absence of intraoperative glucose administration, catabolism had begun to progress by that point. A 1% to 1.5% solution of glucose should, thus, be administered intraoperatively to suppress catabolism. Although blood glucose at M2 was significantly higher than at M1 in the water-only group, the median level was a clinically insignificant 100 mg/dL. The amount and time of preoperative fluid consumption did not correlate with total ketone body levels in either the AW or PW group. The groups each had a median fluid consumption of about 250 mL and median time of consumption of 3.5 to 6 hours before operating room entry. These small intergroup differences likely had no bearing on catabolism.

Our study conducted under strict and realistic management suggests that consuming 250 mL of an 18% carbohydrate beverage from the night before surgery to 2 hours before the start of anesthesia sufficiently inhibits catabolism during anesthesia in minor surgeries of approximately 2 hours duration. Based on these results, consuming an 18% carbohydrate beverage may appear to be better in suppressing lipid and protein catabolism even in minor surgeries. However, the present phase II trial provides preliminary data that need to be confirmed in future phase III trials.

Recently, rapid turnover proteins (RTPs) are being used for evaluating acute-phase nutrition because of their shorter half-life than albumin, which has a 3-week half-life [32]. Tsutsumi and colleagues found no differences in prealbumin levels in patients preoperatively receiving an oral 18% carbohydrate solution or water [19]. With a serum half-life to the order of 2 to 3 days, prealbumin is unlikely to be affected by short-term preoperative fasting [32]. We analyzed the RTP retinol binding protein in this study. The short, 12-hour half-life of retinol binding protein is suitable for measuring the effects of preoperative short-term fasting and carbohydrate loading [32]. However, the levels in the groups at operating room entry (M1) did not differ and were within the normal range and, therefore, did not demonstrate any clear effect of preoperative oral carbohydrate loading. Intra-group comparisons, however, showed that retinol-binding protein levels were significantly lower at M2 as compared to M1 in both AW and PW groups. However, since the levels at both M1 and M2 remained within the normal range, the nutritional significance of this result might be small. Yet, the level would likely have fallen much more with longer anesthesia and in the absence of glucose administration. Nutritional concerns, thus, might indicate the need for intraoperative glucose administration.

Retinol binding protein is one of the negative acute-phase proteins, as are albumin, transferrin and transthyretin. Greater amounts of positive acute-phase proteins (C-reactive protein, serum amyloid A and haptoglobin) are released by hepatocytes following cytokine stimulation in response to trauma or inflammation (acute phase response). The positive acute-phase proteins reportedly play general roles in opsonization and trapping of micro-organisms and their products, and in modulating the host’s immune response [19,33,34]. The physiological reason for decreased synthesis of negative acute-phase proteins is believed to be to conserve amino acids for their utilization in the production of positive acute-phase proteins, enhancing the efficiency of the response [33,34]. In this study, decreased retinol binding protein levels could also have been due to the above mechanism.

The adipocytokines adiponectin and leptin, both of which are factors that increase insulin sensitivity [35–37], were analyzed in this study. Preoperative oral carbohydrate loading in this study was not shown to have had any clear effect on these parameters, which were comparable in the two groups on operating room entry. Levels of both these parameters had declined by M2, suggesting increased insulin resistance. The ERAS program states that preoperative oral carbohydrate loading reduces insulin resistance, but states nothing about its relationship to intraoperative glucose administration [8,9]. Our study indicated that absence of intraoperative glucose administration leads to the onset of catabolism, increases insulin resistance, and possibly causes small decreases in nutritional parameters after 2 hours, suggesting that intraoperative glucose administration at a dose that does not excessively increase blood glucose appears to be necessary.

Our choice to include only minor surgeries in order to exclude the effects of surgical invasiveness and bleeding on metabolism constituted a limitation, in that measurements were possible for only up to 2 hours after the start of anesthesia. Postoperative measurements were not taken because following surgery, the patients were woken, extubated, and postoperatively managed in a conscious state, meaning that increased physical activity would have affected lipid and protein catabolism. Trends in the parameters beyond 2 hours after the start of anesthesia and postoperative parameters are therefore unclear, which is another study limitation. Further research is needed, in which these parameters are measured over a longer time period in patients postoperatively managed at rest in an intensive care unit after minimally invasive surgery.

Another concern is that the Arginaid Water solution, which contains arginine, might have effects on insulin-mediated glucose disposal and might regulate metabolism of the energy substrate [18,19]. However, Tamura and colleagues showed that decrease in insulin sensitivity could be significantly prevented by preoperative loading with 18% carbohydrate (Arginaid Water), similar to the improvement achieved with 12.6% carbohydrate loading using the hyperinsulinemic normoglycemic clamp method. Therefore, clinically, arginine might have only few effects on insulin-mediated glucose disposal [18].

## Conclusions

Participants preoperatively receiving oral carbohydrate loading had lower blood ketone body and FFA levels and higher insulin levels on entry to the operating room. However, in all the participants in this study, none of whom received glucose during the surgery, ketone body and FFA levels rose and insulin levels fell after 2 hours irrespective of whether or not they had received preoperative carbohydrate loading, although ketone body and FFA levels were still lower in the participants preoperatively receiving oral carbohydrate loading. Although retinol binding protein, adiponectin and leptin levels did not differ according to whether oral carbohydrate loading was given preoperatively, the levels of these substances in each group were lower after 2 hours compared with levels on operating room entry, suggesting the possibility of small decreases in nutritional condition and insulin sensitivity.

## Supporting information

S1 Fig Application of Ethical and Scientific Examination of study protocol in English. (DOC)

S2 Fig Application of Ethical and Scientific Examination of study protocol in Japanese. (DOC)

S3 Fig CONSORT 2010 flow Diagram. (DOC)

S1 Table CONSORT 2010 Checklist. (DOC)
