# Supplementary material for: Effects of preoperative oral carbohydrate intake on catabolism, nutrition and adipocytokines during minor surgery: A randomized, prospective, controlled clinical phase II trial
Source: PLoS One. 2019 May 13;14(5):e0216525. doi: 10.1371/journal.pone.0216525 (PMC6513065; doi:10.1371/journal.pone.0216525)
Supplement: S1 Fig — (DOC) [file pone.0216525.s001.doc]

(Style 1)　　　　　　　　　　　　　　　　　　　　　　　　 　* No.

*Date

**Application of Ethical and Scientific Examination of Study Protocol**

Kanagawa Dental University

Research Board

and Ethics Committee

Chairperson: Prof. Yukio Hirata

　　　　　　　　　　　　　　　　　　　　Principle investigator

Department of Critical Care Medicine and Dentistry,

Professor: Yoshinari Morimoto

I approve the principle investigator submits the study protocol described below.

　　　　　　　　　　　　　　　　　　　　Department of Critical Care Medicine and Dentistry,

Professor: Yoshinari Morimoto

We will submit the study protocol described as follows.

| 1.Title | The study of improvement for glucose-lipid metabolism: the effect of preoperative starving protocol for glucose-lipid metabolism |
| --- | --- |
| 2.Principle investigator | Department of Critical Care Medicine and Dentistry,  Professor: Yoshinari Morimoto/ morimoto@kdu.ac.jp |
| 3.Leading research | Department of Critical Care Medicine and Dentistry,  Associate Professor: Uno Imaizumi  Assistant Professor: Chizuko Yokoe  Research Associate: Tomoko Kinugawa |
| 4. **Summary of the study:** In this study, all participants allowed to drink indicated liquids before anesthesia (including clear liquid with/ without carbohydrate in adults; formula or clear liquid with carbohydrate in infants and children). The body composition (total body water, protein, muscle volume, glycogen and body fat volume, et al.) are measured by Bioelectrical Impedance Analysis（BIA）method, and blood test including ketone bodies, blood glucose, free fatty acid (FFA), insulin, rapid turnover protein (RTP；retinol binding protein), leptin, adiponectin and 3-metyl histidine (3-MH) are measured. Then, the purpose of this study is to clarify the effect of the preoperative fasting for glucose-lipid metabolism during anesthesia in infants, children and adults. | |
| 5.Study duration  From approval day of the study protocol by ethics committee to March 31. 2018 | |
| 6.Objectives, case control and implement site  The patients without any metabolic abnormalities (diabetes mellitus, et al.), who underwent oral and maxillofacial surgery (ASA PS 1 or 2).  Case control: 17 patients per group (34 patients in all) in adults.  If the study includes infants and children, 17cases of them are additionally included (total 51 cases). This group is analyzed separately.  Implement site: Kanagawa Dental University Hospital, operating room and ward. | |
| 7．Informed Consent  　　□　none (How to open the outcomes? )  　　☑　Yes　　　☑ document  □ verbal communication (How to record? ) | |
| 8．Manager of the personal information  Department of Critical Care Medicine and Dentistry, Professor: Yoshinari Morimoto | |
| 9．Relevant article 　☑ yes　　　□ no | |

(Style 2)

**Study Protocol**

　1. Study Title: The study of improvement for glucose-lipid metabolism: the effect of preoperative starving protocol for metabolism

　2. Implementation system of study

　　　・Name of affiliation: Department of Critical Care Medicine and Dentistry, Graduate School of Dentistry, Kanagawa Dental University

　　　・Name of Researchers: Department of Critical Care Medicine and Dentistry,

Professor: Yoshinari Morimoto

Associate Professor: Uno Imaizumi

Assistant Professor: Chizuko Yokoe

Research Associate: Tomoko Kinugawa

3. Purpose and meaning

(1) Purpose

The Practice Guidelines for Preoperative Fasting (ASA or JSA) state that patients of any age may ingest formula for up to 6 hours and breast milk for up to 4 hours in infants and children, and clear liquids for up to 2 hours before anesthesia induction. In this study, all participants allowed to drink above liquids before anesthesia (including clear liquid with/ without carbohydrate in adults; formula or clear liquid with carbohydrate in infants and children). The body composition (total body water, protein, muscle volume, glycogen and body fat volume, et al.) are measured by Bioelectrical Impedance Analysis（BIA）method, and blood test including ketone bodies, blood glucose, free fatty acid (FFA), insulin, rapid turnover protein (RTP；retinol binding protein), leptin, adiponectin and 3-metyl histidine (3-MH) are measured. Then, the purpose of this study is to clarify the effect of the preoperative fasting for glucose-lipid metabolism in infants, children and adults.

(2) Meaning

Under physiologic circumstances, glucose and fatty acids are the major blood borne sources of energy for the body. Certain conditions, such as fasting or after heard exercise, mobilize stored glycogen, which is catabolized to produce glucose (gluconeogenesis). In addition, lipolysis is accelerated, and ketone bodies (acetoacetate or β-hydroxybutyrate: β-HBA) are mainly produced through β-oxidation of fatty acids in the liver as an energy source to be used in the brain and peripheral tissues. When the ketone body concentrations are excessively elevated, metabolism can deteriorate to a state of ketoacidosis because of lipid catabolism, representing a nonphysiologic condition (Edmond OB, et al: Diabetes Metab Res Rev 5: 247, 1989; Mitchell GA,et al: Clin Invest Med 18: 193, 1995; Leelanukrom R, et al: Paediatr J Anesth 10: 353, 2000). Preoperative fasting and intraoperative infusion without glucose results in degeneration of lipids and protein from stored glycogen in the liver (Yokoyama T, et al: Asia Pac J Clin Nutr 17: 523, 2008; Yamasaki K, et al: J Anesth 24: 426, 2010).

The Practice Guidelines for Preoperative Fasting (ASA or JSA) state that patients of any age may ingest clear liquids for up to 2 hours before anesthesia induction. This is based on fluctuation of blood glucose to prevent hypoglycemia. Because preoperative liquid intake even without carbohydrate brings no hypoglycemia, it is not considered whether carbohydrate is necessary in the preoperative liquid. Although, it may be better that preoperative liquid and intraoperative infusion contains carbohydrate to depress ketogenesis during surgery, there are few studies to investigate these fields.

Some studies noted that younger children (age <7 years) displayed relatively early onset of active ketogenesis in infants and children, because the volume of glycogen storage is small and gluconeogenesis is poor, lipid mobilization is more likely to occur, lead to ketosis (Nilsson K, et al: Br J Anaesth 56: 375, 1984). In contrast, some studies noted that when starvation is continued in infants and children, children older than 4 years showed a later onset of lipid mobilization owing to sufficient glycogen and gluconeogenetic substrate stores (Bonnefont JP, et al: EurJ Pediatr 150: 80, 1990). Mikawa et al. reported because amount volume of glycogen is stored, ketogenesis is rare with standard preoperative fasting and intraoperative infusion without glucose (Mikawa K, et al: Anesthesiology 74: 1017, 1991).

In adults, blood ketone body concentration elevates under intraoperative infusion without glucose (Yamasaki K, et al: J Anesth 24: 426, 2010). However, there are few studies observing glucose- lipid metabolism with/ without preoperative carbohydrate loading, as same in the elderly. Its objective was to compare changes in intraoperative glucose-lipid metabolism, nutritional parameters and adipocytokines in a group of participants preoperatively receiving an oral carbohydrate solution and a group given water alone, to evaluate the effects and limitations of preoperative oral carbohydrate loading in adult cases.

In this study, the standard starvation (approved by ASA or JSA) is performed preoperatively in adult (≥16 yo), body composition (total body water, protein, muscle volume, glycogen and body fat volume, et al.) using BIA method (BioScan 920- II® system (MP Japan co.)). The items indicating glucose-lipid metabolism (ketone bodies, FFA, insulin, 3-MH, retinol binding protein, leptin and adiponectin), are measured to evaluate the effects and limitations of preoperative oral carbohydrate loading in adult participants. Especially, the comparison between preoperative liquid intake with carbohydrate and plain water is also carried out in adult participants.

Additionally, if infant and child cases can be involved, body composition and items indicating glucose-lipid metabolism are also measured under preoperative liquid intake containing carbohydrate and intraoperative infusion with glucose in infants and children (≤15 yo) as another measurement (this group is analyzed separately).

　4. Methods and duration of the study

　(1) Methods:

1) Each participant is informed about the prospective study protocol using the full explanatory note, and a written informed consent is obtained.

2) The patients who agree with the study protocol are registered, and information listed below are obtained.

　　1. age

　　2. sex

　　　3. height

　　　4. weight

　　　5. preoperative laboratory data: blood (white cells, red cells, hemoglobin, hematocrit, platelet count), biochemical test (total protein, albumin, urine nitrogen, creatinine, total bilirubin, AST, ALT, LDH, ALP, γ-GTP, choline esterase, amylase, CK, blood glucose, total cholesterol, lipid, sodium, potassium, chloride), and urine test (urine glucose, urine ketone body, urine protein)

3) The participants underwent BIA method at evening (between 16:00 and 22:00) to evaluate body composition on the day before surgery (measurement is performed about 10 seconds).

4) The standard starvation (approved by ASA or JSA) is performed preoperatively in all participants (formula for up to 5 hours and clear liquid for up to 2 hours).

1. Adult groups

The participants are allowed to consume either beverage with carbohydrate (Arginaid water®, Nestle Health Service: AW) or plain water (PW) from 9:00 p.m. on the day before surgery to 7:00 a.m. on the day of surgery, based on their randomization. The volume remaining is measured in a graduated cylinder to determine the volume consumed.

2. Infants and children group

For preoperative liquid intake, the infants and children are allowed to drink formula without any volume restriction for <6 hours before entering the operating room and glucose solution (Arginaid water® (AW)) or Pocari Sweat®) for <2 hours before entering the operating room. These glucose containing liquids are selected ethically based on minimizing the risk of ketogenesis in infants and children. Because the amount of breast milk consumption can not be quantified, the infants taking breast milk are excluded from this study.

5) Anesthesia

(1) Adult groups

The patients are brought into the operating room. Then, the electrodes of the BioScan 920-II® are attached, and the first body composition measurement is taken. With peripheral vein access gained via a 20 G or 22G indwelling needle inserted, the first blood sample (M1; 5mL) is collected, following which non-glucose solution is delivered. General anesthesia is induced with an intravenous infusion of 2 μg/kg of fentanyl followed by boluses of 2 mg/kg of propofol and 0.6 mg/kg of rocuronium bromide. Mask ventilation with 3% to 5% sevoflurane or 3% to 8% desflurane in oxygen is given. Nasotracheal intubation is performed in a normal method. The second body composition measurement and blood sampling (M2) are performed 2 hours after the induction of anesthesia.

(2) Infants or children group

Anesthesia is induced under mask ventilation with 3% to 5% sevoflurane in oxygen is given. The electrodes of the BioScan 920-II® are attached after induction, and the first body composition measurement is taken. With peripheral vein access gained via a 22G or 24G indwelling needle inserted, the first blood sample (M1; ketone body, FFA, blood glucose, insulin, retinol binding protein: 3mL) is collected, following glucose containing solution is delivered. Nasotracheal intubation is performed in a normal method with an intravenous infusion of 0.6-1.0 mg/kg of rocuronium bromide. The 1% glucose solution is delivered with infusion rate of 120 -200mg/kg/h of glucose (Leelanukrom R, et al: Paediatr J Anesth 10: 353, 2000). Normal anesthesia management is performed. If possible, an arterial blood analysis is performed during anesthesia.

The second body composition measurement and blood sampling (M2) are performed 2 hours after the induction of anesthesia (3cc of blood volume).

6) Blood test and body composition measurement in M1 and M2

・**Blood test items:** ketone bodies, FFA, blood glucose, insulin, 3-MH, retinol binding protein, leptin and adiponectin in adults. The ketone bodies, FFA, blood glucose, insulin, retinol binding protein are measured in infants and children.

・**Items measured by BIA method:** resting metabolic rate: RMR, fat removal quantity, body cell mass, fat removal rate, body fat percentage, total body water, total body water percentage, extra cellular water volume, intra cellular water volume, extra cellular water percentage, intra cellular water percentage, extracellular mass, protein volume、mineral volume, muscle volume, total potassium volume, total calcium volume, glycogen volume, dry weight, extracellular solid, extra cellular fluid, plasma volume, body mass index, body density

・Blood sample is delivered to the outside laboratory company to measure each item.

7) Evaluation of outcomes

Primary：Comparison of metabolic data (blood ketone body, FFA, blood glucose, insulin, retinol binding protein, leptin, adiponectin, 3-MH) and body composition measured by BIA method between preoperative carbohydrate loading cases and non-loading cases in adults.

Secondary：The data of above items in infants or children are analyzed if data can be collected from the infants and children group.

8) Number of cases

　　　 When determining the required number of patients in adults, we found that a sample size of 14 patients per group would provide a power of 80% at an alpha error of 0.05 and beta error of 0.20, based on the findings in a comparison of total blood ketone bodies in patients receiving intravenous intraoperative fluids with and without 1% glucose We ultimately determined that 17 patients per group (34 patients in all) would be necessary, assuming a 20% dropout rate.

As all of infants and children drink liquid containing carbohydrate and intraoperative infusion containing glucose, sample size is not calculated; seventeen patients are necessary as same as in adults. These data are suggested as separate data from data in adult groups, because the way of administrating glucose is different between in adult groups and in infant and child group.

9) Ethics

In this study, all anesthetic procedures are performed within standard methods, and no additional invasive procedures are employed to participants. Therefore, all procedures are as safe as other anesthesia without ethical problems. The body composition analysis is performed using impedance method with attachment of 8 seats of electrodes like ECG examination; there are no invasive procedures to participants. Then, 10cc of blood sampling in adults, and 6cc in infants or children bring no insult to participants.

5. Schedule and duration

(1) Study schedule

　　　　　　 　 　 　 Ward　　　　　　　　 　　　Operating room

Time schedule

　　　　　　　　　　 Preope. day　　　　 Op. day

General anesthesia (preope. round)　 　 　starving　　　　　general anesthesia

Study　　　　　 measurement of body　　　　 　　　　　　blood sampling (2 times)

composition by BIA (adults: 5mL each, infants and children 3mL each)

　　　　　　　　　　　　　　　　　　measurement of body composition

　(2) Study duration

From approval day of the study protocol by ethics committee to March 31, 2018.

6. Inclusion and exclusion criteria

Inclusion criteria:

　 (1) The patients without any metabolic abnormalities (diabetes mellitus, et al.), who underwent oral and maxillofacial surgery under general anesthesia (ASA PS 1 or 2).

　 (2) The patients who understand the study protocol and agree with it. The proxy can consent with the study when the patients are under 15 years of age.

　Exclusion criteria

(1) The patients who has poorly controlled general complication (ASA PS ≥3)

(2) The patients with metabolic abnormalities (diabetes mellitus, et al.)

(3) The patients who are considered as inappropriate for the study by researchers

　Criteria of suspension

　 (1) The patients whose condition deteriorates during general anesthesia

(2) The scheduled volume of sampling blood cannot be gained by any reason

(3) The patients who revoke the consent

6. Scientific rationale of the study

The Practice Guidelines for Preoperative Fasting state that patients of any age may ingest clear liquids for up to 2 hours before anesthesia induction. This is based on attenuation of blood glucose to prevent lowering blood glucose level. Although, it may be better that preoperative liquid contains carbohydrate to depress ketogenesis during surgery, there is few study to investigate these fields. Then, as there are little studies to investigate in relation of preoperative fasting to glucose-lipid metabolism in any age, constant findings are not established.

In this study, there are no additional burdens to participants because normal preoperative fasting and anesthesia management are performed. Based on the outcomes of preoperative fasting (comparison between carbohydrate loading or not in adults), the relation between preoperative fasting and glucose- lipid metabolism is become clear, and these findings may lead to change the guideline.

　7. Procedure of the informed consent

1) **☑**Study with obtaining new patients sample and data

Informed consent is obtained from proxy when the participant is under 15 years of age. The proxy is generally a person in parental authority or guardians of minor. The principal investigator or leading research explain the study contents concisely to participants or proxies using explanatory notes. At that time, the opportunity of asking and enough time for judgement whether to join or not to join the study should be provided to the participants or proxies. It depends on the spontaneous decision of participants or proxies whether to join the study.

The researchers obtain the informed consent from the participants in consideration with followed items; the participants receive no advantage or disadvantage in the clinical diagnosis and treatment procedure whether they participate the study or not. The participants also can withdraw the consent after accepting the consent at once with no disadvantage. When the participants or proxies give informed consent spontaneously, they bear their signature and the date in the informed consent form. The researchers give their name and the date when they explain the study. When they cancel their consent after giving consent at once, they fill out the withdrawal form. When the explanatory notes or consent form are revised, researchers reacquire the consent.

　　2) **□**Study with possessing patients sample and data in the affinity

　　3) **□**Case with providing patients sample and data to other institute

　　4) **□**Study with receiving patients sample and data from other institute

　8. Management of the personal information

As the data obtaining from participants include patient’s personal information such as name and diagnosis, the handling of the data should be noticed. To prevent lose data or identify the personal information, some measures are thoroughly employed as described follows.

　　　The participant’s name, birth date, patient’s number, address and telephone number in the sampling blood and clinical information are deleted and the participants are anonymized using registered number. Both the participant and registered number are linked by corresponding list; the corresponding file is installed in the stand-alone PC and security key words are set up. The principal investigator has the key of the room where this PC is set, and manages the room security. The manager of the personal information is Prof. Yoshinari Morimoto (Department of Critical Care Medicine and Dentistry).　The sampling blood is delivered to outside laboratory company after anonymization.

9. Burden and predicted risk or benefit for the participant, and the measures to minimize these burden or risk

　　(1)　**Burden for participants:** Ten mL of sampling blood volume in adults and 6 mL in infants and children are collected.

　　(2)　**Predicted risk and benefit:** Participants directly receive no benefit from this study.

The volume of sampling blood is low risk with no problem for health. The participants receive no stress because blood is collected when IV line is established or during anesthesia.

　　(3)　**Total evaluation:** The participants receive little burden and risk.

　　(4)　**The measures to minimize these burden or risk:** The participants receive no stress because blood is collected when IV line is established or during anesthesia, or sampling blood during anesthesia is performed at the same time of ordinary blood test. When any problems are occurred during sampling blood, the study is suspended and the doctor in charge treats the participants.

10. How the samples and information (including relevant information of the study) are stored or disposed after completion of the study?

　　1) Storage: the samples and information collected in this study will be stored for 5 years after completing the study at Kanagawa Dental University, Department of Critical care Medicine and Dentistry (under responsibility of Prof. Yoshinari Morimoto).

　　2) Dispose: the samples and information will be disposed as medical waste after deleting the registered number. These data will not be reused in other studies in the future.

11. Report to the affiliation heads

　　Progression of the study will be reported to the affiliation head annually or when the study is ended.

12. Funding of the study, COI of affiliation and researchers, and benefit from this study

　　1) Funding

**□** Departmental funding

**☑** A grant from the Grants-in-Aid for Scientific Research (Scientific Research C)

**□** others (　　　　　 　)

　　2) Conflict of Interest (COI)

**□** The committee accepted the COI findings.

**□** It was reported to the committee.

　　　☑ No.

13. How to open the study outcome?

The outcome of this study will be submitted to the international or Japanese scientific meeting and journal. The information being able to detect individual must not be contained at that time.

14. Consultation from the participants or proxies

　　The participants or proxies can consult with the principal investigator Prof. Yoshinari Morimoto (Department of Critical Care Medicine and Dentistry) about this study.

15. Procedures of obtaining the informed consent by the participants or proxy

　　1) **□** none

　　2) **☑** yes (Researchers explain the study contents to participants or proxies using explanatory notes and obtain the written informed consent. When the participants are under 15 years of age or who cannot judge whether to participate the study, the informed consent is submitted by the proxy and participant, if possible.)

16. Procedures of obtaining the informed assent

　　1) **□** none

　　2) **☑** yes (Researchers explain the study contents to participants or proxies using explanatory notes and obtain the written informed consent. When the participants are under 15 years of age or who cannot judge whether to participate the study, the informed consent is submitted by the proxy and participant, if possible.)

17. How do the researchers judge the situation that is fully adapted to Ethical guideline: Article 12-5, when the participants are in the emergent and obvious life-threating conditions.

　　1) ☑ none (methods for judgement:

　　2) □ yes (contents：　　　　　　　　　　　　　　　　　　　　　　)

18. How is the economic burden or reward of participants?

1) economic burden　☑ none　(The necessary charges of anesthesia and operation et al. are payed)

**□** yes (contents：　　　　　　　　　　　　　　　　　　　　　　)

2) reward 　☑ none

**□** yes (contents：　　　　　　　　　　　　　　　　　　　　　　)

19. Invasion of the study if applicable (excluded of minor invasion)

　　1) **☑** none

　　2) **□** yes (contents：　　　　　　　　　　　　　　　　　　　　　　)

20. The compensation for health damage caused by the invasive methods of the study

　　1) **☑** none

　　2) **□** yes (contents：　　　　　　　　　　　　　　　　　　　　　　)

21. Do the participants receive medical practice when the large medical invasion beyond the normal medical practice is performed in the study?

　　1) **☑** none

　　2) **□** yes (contents：　　　　　　　　　　　　　　　　　　　　　　)

22. How are the study outcomes of participants (including the episodic findings) managed when health of the participants or successive hereditary features are possibly affected by the study?

　　1) **☑** none

　　2) **□** yes (contents：　　　　　　　　　　　　　　　　　　　　　　)

23. How do the researchers supervise the contents of the study and trustees when some parts of the study are consigned?

　　1) **□** none

　　2) **☑** yes (the measurements of sampling blood are ordered to the outside laboratory company. The sampling blood is delivered to the laboratory company after anonymization at Kanagawa Dental University. )

24. Does the sample or information collected from participants possibly reuse for another studies that cannot be identified when participants consent the study?

Does the sample or information possibly provide to another institute?

　　1) **☑** none

　　2) **□** yes (contents：　　　　　　　　　　　　　　　　　　　　　　)

25. How are the implementation and operation procedure decided when monitoring and inspection of the study based on the Ethical Guideline: article 20 is performed?

　　1) **☑** none

　　2) **□** yes (contents：　　　　　　　　　　　　　　　　　　　　　　)

(Style 3)

**Explanatory Note of the Study**

1. Study Title: The study of improvement for glucose-lipid metabolism: the effect of preoperative starving protocol for metabolism

　This study is approved by the institutional head.

2. Research system of the study

・Name of affiliation: Department of Critical Care Medicine and Dentistry, Graduate School of Dentistry, Kanagawa Dental University

　Principle investigator: Prof. Yoshinari Morimoto, Department of Critical Care Medicine and Dentistry,

3. Purpose and meaning

(1) Purpose

The patients of any age are starved for up to several hours before anesthesia, and ketone bodies are mainly produced because of shortage of carbohydrate when normal starvation is performed.

Under physiologic circumstances, carbohydrate (glucose) is the major blood borne sources of energy for the body. Certain conditions, such as fasting or after exercise, body components are catabolized to produce ketone bodies excessively, those lead to abnormal metabolism becoming systematic disease. Although, it may be better that preoperative liquid and intraoperative infusion contains carbohydrate to depress ketosis during surgery, there are few studies to investigate these metabolism and treatment.

In this study, the standard starvation is performed preoperatively in adults, infants and children, body composition (total water volume, protein, et al.) using bioelectrical impedance analysis (BIA) method (BIA measures the resistance (impedance) to passage of a weak, harmless electrical current through the body). The items indicating glucose-lipid metabolism (ketone bodies, blood glucose, et al.) are measured to evaluate the effects of preoperative oral carbohydrate loading in all aged participants. Then, the purpose of this study is to clarify the effect of the preoperative fasting for glucose-lipid metabolism in infants, children and adults. Both preoperative intake and intraoperative infusion contain glucose in infants and children.

(2) Meaning

The abnormal metabolism caused by preoperative starvation becomes clear based on the results of this study, and the appropriate treatments (including preoperative liquid intake or intraoperative fluid infusion) can be developed.

4. Methods and duration of the study

(1) Methods:

The healthy patients without any metabolic abnormalities (diabetes mellitus, et al.), who underwent oral and maxillofacial surgery in our hospital are included in this study. On the other hand, the patients with any metabolic abnormalities are excluded. There is no additional burden for the patients, because normal anesthesia is carried out without special intervention.

When the patients joined this study, body composition measurement and blood sampling (total of 10 mL in adults and 6 mL in infants and children) are performed to investigate intraoperative metabolic conditions (ketone bodies, blood glucose, et al) as well as body composition.

1) The participants undergo BIA method at evening (between 16:00 and 22:00) to evaluate body composition on the day before surgery (measurement is performed about 10 seconds). The BIA is performed using impedance method with attachment of 8 seats of electrodes like ECG examination

2) The standard starvation is performed preoperatively in all participants (formula for up to 6 hours and clear liquid for up to 2 hours) on the day of surgery. The volume remaining is measured in a graduated cylinder to determine the volume consumed.

1. Adult groups

The participants are allowed to consume either beverage with carbohydrate or plain water (PW) from night 9:00 p.m. on the day before surgery to 7:00 a.m. on the day of surgery.

2. Infants and children group

For preoperative liquid intake, the infants and children are allowed to drink formula without any volume restriction for <5 hours before entering the operating room and glucose solution for <2 hours before entering the operating room. The volume of intake is measured by feeding bottle or graduated cylinder. Because the amount of breast milk consumption could not be quantified, the infants taking breast milk are excluded from this study.

3) The adult patients are brought into the operating room. The electrodes of the BioScan 920-II® are attached, and the first body composition measurement is taken. With peripheral vein access gained, the first blood sample (M1: 5mL) is collected, and standard anesthesia is carried out. The intraoperative infusion without glucose is performed.

4) In infants or children, anesthesia is induced under inhalation anesthesia gas, followed by the first body composition measurement. With peripheral vein access gained, the first blood sample (M1; ketone body, FFA, blood glucose, insulin, retinol binding protein: 3mL) is collected, and standard anesthesia is carried out including intraoperative infusion with glucose.

5) General anesthesia is performed in standard method. The second body composition measurement and blood sampling (M2: 5mL in adults, and 3mL in infants and children) are performed 2 hours after the start of anesthesia. When possible, arterial blood gas analysis is performed.

* Blood test and body composition measurement in M1 and M2

1)・Blood test items: ketone bodies, free fatty acid, blood glucose, insulin, 3-methl hustidine, retinol binding protein, leptin and adiponectin in adults. The ketone bodies, free fatty acid, blood glucose, insulin are measured in infants and children.

・Body composition measured by BIA method

・Blood sample is delivered to the outside laboratory company to measure each item.

2) Study schedule

　　　　　　 　 　 　 Ward　　　　　　　　 　　　Operating room

Time schedule

　　　　　　　　　　 Preope. day　　　　 Op. day

General anesthesia (preope. round)　 　 　starving　　　　　general anesthesia

Study　　　　　 measurement of body　　　　 　　　　　　blood sampling (2 times)

composition by BIA (adults: 5mL each, infants and children 3mL each)

　　　　　　　　　　　　　　　　　　measurement of body composition

　(2) Study duration

From approval day of the study protocol by the ethics committee to March 31. 2018.


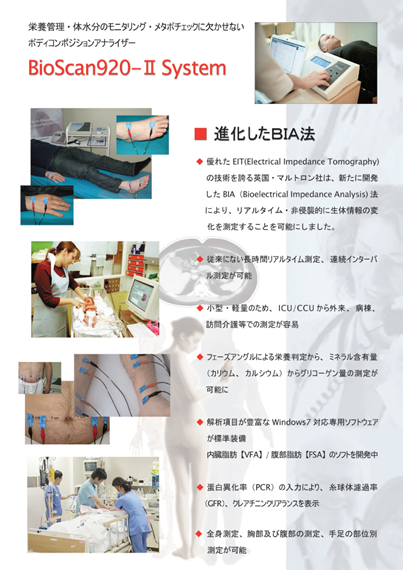


5. Why are you selected as participant?

The healthy patients without any metabolic abnormalities (diabetes mellitus, et al.), who undergo oral and maxillofacial surgery in our hospital are included in this study.

6. Burden and predicted risk or benefit for the participant, and the measures to minimize these burden or risk

(1)　**Burden for participants:** Ten mL of sampling blood volume in adults and 6 cc in infants and children. The volume of sampling blood is low with no problem for health. The participants receive no stress because blood is collected when IV line is established or during anesthesia.

(2)　**Predicted risk and benefit:** Participants directly receive no benefit from this study.

**The measures to minimize these burden or risk:** The participants receive no stress because blood is collected when IV line is established or during anesthesia, or sampling blood during anesthesia is performed at the same time of ordinary blood test. When any problems are occurred during sampling blood, the study is suspended and the doctor in charge treats the participants.

7. Procedure of the informed consent

The informed consent depends on the participants or proxies spontaneous decision whether to join the study. The researchers obtain the informed consent from the participants in consideration with followed items; the participants receive no advantage or disadvantage in the clinical diagnosis and treatment procedure whether they join the study or not. The participants also can withdraw the consent after giving the consent at once with no disadvantage. In that case, collected blood and results are disposed, and patient information is not used in this study. However, it is impossible to cancel the consent after the results have already published to journal or scientific meeting, or the participant information is not detected because they were anonymized totally when a participant declares to withdraw the consent. The volume of sampling blood is small and there is no problem for the health. When any problems are occurred during sampling blood, the study is suspended and the doctor in charge treats the participants.

9. How to open the study outcome?

The outcome of this study will be submitted to the international or Japanese scientific meeting and a journal. The information being able to detect individual must not be contained at that time.

10. How to disclose the study protocol?

　　The information of this study protocol can be inspected when the participants and proxies request. The participants or proxies can ask to the principal investigator.

11. Management of the personal information

As the data collecting from participants include patient’s personal information such as name and diagnosis, the handling of the data should be noticed. To prevent lose data or identify the personal information, some measures are thoroughly employed as described follows.

　 The participant’s name, birth date, patient’s number, address and telephone number in the sampling blood and clinical information are deleted and the participants are anonymized using registered number. The participant and registered number are linked by corresponding list; the corresponding file is installed in the stand-alone PC and security key words are set up. The principal investigator has the key of the room where this PC is set, and manages the room security. The manager of the personal information is Prof. Yoshinari Morimoto (Department of Critical Care Medicine and Dentistry).　The sampling blood is delivered to an outside laboratory company after anonymization.

12. How the samples and information (including relevant information of the study) are stored or disposed after completion of the study?

　1) Storage: the samples and information collected in this study will be stored for 5 years after completing the study at Kanagawa Dental University, Department of Critical care Medicine and Dentistry (under responsibility of Prof. Yoshinari Morimoto).

　2) Dispose: the samples and information will be disposed as medical waste after deleting the registered number. These data will not be reused in other studies in the future.

13. Funding of the study, COI of affiliation and researchers, and benefit from this study

The cost for this study is supported by a grant from the Grants-in-Aid for Scientific Research. Participants should pay only the cost for medical treatments.

　　1) Funding

**□** Departmental funding

**☑** A grant from the Grants-in-Aid for Scientific Research (Scientific Research C)

**□** others (　　　　　 　)

　　2) Conflict of Interest (COI)

**□** The committee accepted the COI findings.

**□** It was reported to the committee.

　　　☑ No.

14. Consultation from the participants or proxies

　　The participants or proxies can ask to the principal investigator Prof. Yoshinari Morimoto (Department of Critical Care Medicine and Dentistry) about this study.

15. How is the economic burden or reward of participants?

1) economic burden　☑ none　(The necessary charges of anesthesia and operation et al. are payed)

**□** yes (contents：　　　　　　　　　　　　　　　　　　　　　　)

2) reward 　☑ none

**□** yes (contents：　　　　　　　　　　　　　　　　　　　　　　)

16. Do the participants receive medical practice when the large medical invasion beyond the normal medical practice is performed in the study?

Items about other treatment:

　　1) **☑** none

　　2) **□** yes (contents：　　　　　　　　　　　　　　　　　　　　　　)

Providing medical practice to the participants after the study is completed:

　　1) **☑** none

　　2) **□** yes (contents：　　　　　　　　　　　　　　　　　　　　　　)

17. How are the study outcomes of participants (including the episodic findings) managed when health of the participants or successive hereditary features are possibly affected by the study?

　　1) **☑** none

　　2) **□** yes (contents：　　　　　　　　　　　　　　　　　　　　　　)

18. Invasion of the study

　　1) **☑** none

　　2) **□** yes (contents：　　　　　　　　　　　　　　　　　　　　　　)

19. Does the sample or information collected from participants possibly reuse for other studies that cannot be identified when participants consent the study?

　　1) **☑** none

　　2) **□** yes (contents：　　　　　　　　　　　　　　　　　　　　　　)

20. Invasion of the study with intervention (excluded of minor invasion)

　　1) **☑** none

　　2) **□** yes (contents：　　　　　　　　　　　　　　　　　　　　　　)
