# Supplementary material for: Effects of preoperative oral carbohydrate intake on catabolism, nutrition and adipocytokines during minor surgery: A randomized, prospective, controlled clinical phase II trial
Source: PLoS One. 2019 May 13;14(5):e0216525. doi: 10.1371/journal.pone.0216525 (PMC6513065; doi:10.1371/journal.pone.0216525)
Supplement: S2 Fig — (DOC) [file pone.0216525.s002.doc]

（様式１）　　　　　　　　　　　　　　　　　　　　　　　　　※ 受付番号　第　　　　　番

- 受付日　　年　　月　　日

**研究倫理審査申請書**

学校法人神奈川歯科大学

研究倫理審査委員会

　委員長　平田　幸夫　　殿

　　　　　　　　　　　　　　　　　　　　　　（実施責任者）所属　全身管理医歯学講座

　　　　　　　　　　　　　　　　　　　　　　　　　　　　　職名　教授

　　　　　　　　　　　　　　　　　　　　　　　　　　　　　氏名　森本　佳成　　　　　　　㊞

上記の者が、貴委員会に下記研究について実施の申請をすることを承認します。

　　　　　　　　　　　　　　　　　　　　 (講座等の担当教授)所属　全身管理医歯学講座

　　　　　　　　　　　　　　　　　　　　　　　　　　　　　氏名　森本　佳成　　　　　　　㊞

下記事項について、倫理審査を申請いたします。

| 1.課題名 | 周術期における糖脂質代謝の改善に関する研究―術前の絶飲絶食が糖脂質代謝に及ぼす影響について |
| --- | --- |
| 2.実施責任者 | 所属・職名・氏名・連絡先(内線・e-mail)  全身管理医歯学講座・教授・森本佳成・2259/ morimoto@kdu.ac.jp |
| 3.分担研究者 | 所属・職名・氏名  全身管理医歯学講座・講師・今泉うの  全身管理医歯学講座・助教・横江千寿子  全身管理医歯学講座・医員・衣川智子 |
| 4.研究等の概要　手術に際し術前絶飲絶食を行い麻酔中の血中ケトン体量を測定すると、乳幼児や小児ではケトン体量の異常な上昇がみられるのに対し、成人では大幅な上昇は見られないといわれる。しかし、術前の絶飲絶食による糖脂質代謝への影響を検討した報告はない。本研究の目的は、乳幼児・小児から成人までの各年齢層の被験者の手術に際し、通常の術前絶飲絶食（成人においては、ブドウ糖の投与の有無の比較を含む）を行い、Bioelectrical Impedance Analysis（BIA）法を用いて、被験者の体水分量、蛋白量、グリコーゲン量などの体成分の変化を測定するとともに、血液中の糖脂質代謝の指標となる項目（ケトン体、遊離脂肪酸、血糖、インスリン、レチノール結合蛋白、レプチン、アディポネクチン、3-メチルヒスチジン）を測定し、従来の術前絶飲絶食が糖脂質代謝へ与える問題点を解明する。 | |
| 5.研究期間　　　　　研究倫理審査委員会承認日～　平成30年3月31日 | |
| 6.研究等の対象・予定症例数及び実施場所  ASA　Physical status 1または2の被験者。ただし、糖脂質代謝に影響を与える疾患（糖尿病など）を有する被験者は除外する。  予定症例数51例：成人の比較では各群17例（合計34例）。乳児・小児を行う場合は17例を追加するため合計51例となる（別に統計解析を行う）。  実施場所：神奈川歯科大学附属病院　手術室、麻酔科外来、病棟 | |
| 7．インフォームド・コンセント取得の有無  　　□　無（情報公開の方法：　　　　　　　　　　　　　　　　　　　　　　　　　　）  　　☑　有　　　☑①文書　　□②口頭（記録の方法：　　　　　　　　　　　　　　　） | |
| 8．個人情報管理責任者所属・職・氏名  全身管理医歯学講座・教授・森本佳成 | |
| 9．研究課題に関する研究論文等の写し　☑有　　　□無 | |

※印は研究倫理審査委員会で記入

○添付資料

研究計画書、説明文書、同意書、同意撤回書

　　　　（提出書類はA4縦　横書きにすること）

(様式2)

**研究計画書**

　１．研究の名称：周術期における糖脂質代謝の改善に関する研究―術前の絶飲絶食が糖脂質代謝に　及ぼす影響について

　２．研究の実施体制

　　　・研究機関の名称：神奈川歯科大学大学院歯学研究科全身管理医歯学講座麻酔学分野

　　　・研究者氏名：全身管理医歯学講座・教授・森本佳成

全身管理医歯学講座・講師・今泉うの

全身管理医歯学講座・助教・横江千寿子

全身管理医歯学講座・医員・衣川智子

　３．研究の目的及意義

　（1）目的：乳児・小児から成人までの各年齢層の被験者の手術に際し、米国麻酔科学会および日本麻酔科学会のガイドラインが定める術前絶飲絶食（人工乳は麻酔導入5 時間前まで、母乳は4 時間前まで、clear liquid は2 時間前まで許可；成人においては、ブドウ糖投与の有無の比較を含む）を行い、Bioelectrical Impedance Analysis（BIA）法を用いて被験者の体水分量や蛋白質量、筋肉量、グリコーゲン量、体脂肪量などを測定するとともに、血液中の糖脂質代謝の指標となる項目[ケトン体、血糖、遊離脂肪酸、インスリン、rapid turnover protein（RTP；レチノール結合蛋白）、レプチン、アディポネクチン、3-メチルヒスチジン]を測定し、各年齢群における従来の術前絶飲絶食および術前輸液が糖脂質代謝へ与える影響および問題点を解明することが目的である。

（2）意義：一般に生理的な状態では、ブドウ糖と脂肪酸は、体の主なエネルギー源となる。しかし、絶飲絶食や激しい運動後では、ブドウ糖の摂取不足により、貯蔵されたグリコーゲンが利用され、異化を受けてブドウ糖を産生する（糖新生）。さらに、これら貯蔵グリコーゲンが利用された後は、脂質の動員が加速し、肝臓において脂肪酸のβ酸化によってケトン体（βヒドロキシ酪酸およびアセト酢酸）が産生され、脳や末梢組織においてエネルギー源として利用される。しかし、この状態が持続すると、ケトン体濃度は過度に上昇しケトーシスをきたし、さらには、ケトアシドーシスをきたす危険性が生じる（Edmond OB, et al: Diabetes Metab Res Rev 5: 247, 1989; Mitchell GA,et al: Clin Invest Med 18: 193, 1995; Leelanukrom R, et al: Paediatr J Anesth 10: 353, 2000）。また、術前の絶飲絶食やブドウ糖を含まない輸液により脂質や蛋白質の分解は促進され、肝臓におけるグリコーゲンの貯蔵量が減少する（Yokoyama T, et al: Asia Pac J Clin Nutr 17: 523, 2008; Yamasaki K, et al: J Anesth 24: 426, 2010）。

現在、世界中で広く普及している米国麻酔科学会および日本麻酔科学会の「術前の絶飲絶食ガイドライン」では、clear liquid は2 時間前まで許可されているが、これは血糖値の推移（低血糖を防止する）を基に作成されたもので、ブドウ糖を含有していないclear liquidを飲用しても低血糖は生じないとされるため、ブドウ糖の含有の有無は全く問われていない。しかし、糖脂質代謝（ケトーシスを予防する）を考慮すると、術前に投与するclear liquidまたは輸液には、ブドウ糖を添加するほうがよい可能性が高いと考えられる。

過去の報告では、7 歳未満の乳児や小児では貯蔵グリコーゲン量が少ないため、長時間の絶飲絶食による低血糖により早期に脂質動員がおこり、ケトン体産生が増加し、ケトーシスの危険性が増加するとの報告がある（Bonnefont JP, et al: EurJ Pediatr 150: 80, 1990; Nilsson K, et al: Br J Anaesth 56: 375, 1984）。一方、4 歳以上の小児では十分な量のグリコーゲンが貯蔵されているため、通常の術前絶飲絶食を行い、術中はブドウ糖を含まない輸液を行ってもケトン体産生は見られないことから、ブドウ糖投与の必要性はないとの報告（Mikawa K, et al: Anesthesiology 74: 1017, 1991）があり、一定の見解は得られていない。成人については、術前のブドウ糖投与に関する報告はなく、術中にブドウ糖を含有した輸液とブドウ糖を含有しない輸液を行った比較では、後者で術中に血中ケトン体濃度が有意に上昇するとの報告がある（Yamasaki K, et al: J Anesth 24: 426, 2010）。しかし、術前の絶飲絶食にブドウ糖の投与の是非に関する報告はなく、糖脂質代謝を観察した報告もない。これは高齢者についても同様であり、解明されていない問題である。

本研究では、①　乳幼児（0歳～5歳）、小児（6歳～15歳）、成人（16歳以上）の被験者の周術期に、米国麻酔科学会および日本麻酔科学会のガイドラインが定める術前絶飲絶食（人工乳は麻酔導入5 時間前まで、母乳は4 時間前まで、clear liquid は2 時間前まで許可）を行い、Bioelectrical Impedance Analysis（BIA）法【ボディコンポジションアナライザーBioScan 920-Ⅱ system（エムピージャパン株式会社）を使用】を用いて乳幼児の体水分量や蛋白質量、筋肉量、グリコーゲン量、体脂肪量などを測定するとともに、血液中の糖脂質代謝の指標となる項目［ケトン体、血糖、遊離脂肪酸、インスリン、rapid turnover protein（RTP；レチノール結合蛋白）、レプチン、アディポネクチン、3-メチルヒスチジン］を測定し、各年齢群における従来の術前絶飲絶食および輸液が糖脂質代謝へ与える影響および問題点を解明する。また、②　成人の被験者に与えるclear liquidを、ブドウ糖を含有しない飲料と含有する飲料の2群に分類し、術前のブドウ糖投与の有無が糖脂質代謝へ与える影響および問題点を解明する。乳幼児・小児（≦15歳）では、術前飲料および術中輸液はブドウ糖を含有するものを用いる（成人とは別に分析する）。

　４．研究の方法及び期間

　（1）方法：

１）被験者に別添説明文書に基づき説明を行った上で、本研究（前向き研究）への参加について、文書にて同意を得る。

２）同意が得られた患者を被験者として登録し、登録時に下記の臨床情報を診療録より取得する。

　　① 年齢

　　② 性別

　　　③ 身長

　　　④ 体重

　　　⑤ 通常の術前血液・尿検査値：末梢血液検査（白血球数、赤血球数、ヘモグロビン、ヘマトクリット、血小板数）、生化学検査（総蛋白量、アルブミン量、尿素窒素、クレアチニン量、尿酸、総ビリルビン、AST、ALT、LDH、ALP、γGTP、コリンエステラーゼ、アミラーゼ、CK、空腹時血糖、総コレステロール、中性脂肪、ナトリウム、カリウム、クロール、）、尿検査（尿糖、尿ケトン体、尿蛋白）

３）全身麻酔前日の夕方（16 時〜22 時）に、日常の体内成分値として、被験者にBioScan 920-Ⅱ systemの電極を貼付し、体内成分値の測定を行う（10 秒程度で測定可能）。

４）手術当日、被験者の術前に、米国麻酔科学会および日本麻酔科学会のガイドラインが定める術前絶飲絶食（人工乳は麻酔導入5 時間前まで、clear liquid は2 時間前まで許可）を行う。

①　乳幼児・小児では、飲水量は目盛付きカップまたは哺乳瓶にて測定する。なお、母乳しか飲めない患児は本研究に含めない。

なお、乳幼児・小児では、clear liquidとしてはブドウ糖を含有するアイソカルアルジネードウオーター（Nestle Health Science）またはポカリスエット（大塚製薬）を使用する。これは、乳幼児においては貯蔵グリコーゲン量が少なく、ブドウ糖の投与がないと短時間でケトーシスの危険性があるとの報告もあることから、倫理的な面からの配慮である。

②　成人に対しては、無作為（封筒法）にclear liquidとして、ブドウ糖を含有しないもの（ミネラルウオーター）と含有するもの（アイソカルアルジネードウオーター）の2群に割り付けて投与する。

５）成人：手術室入室後、通法通り20Gまたは22G留置針にて静脈路を確保し、この時に1回目の採血（採血量5.0cc）を行った後、非ブドウ糖含有液（生理食塩水等）にて輸液を開始する。同時に、BioScan 920-Ⅱ systemの電極を貼付し、体内成分値の測定を行う。フェンタニル2µg/kgおよびプロポフォール2mg/kgにて急速導入し、酸素および3～5%セボフルランまたは3～8%デスフルランにてマスク換気を行う。ロクロニウム臭化物0.6～1.0mg/kg にて筋弛緩効果を得た後、通法通り気管挿管を行う。

６）乳幼児・小児では、酸素および3～5%セボフルランを用いてマスク換気により緩徐導入を行い、通法通り22Gまたは24G留置針にて静脈路を確保する。この時に1回目の採血（採血量：3.0cc）を行った後、1%ブドウ糖含有液（フィジオ140）にて輸液を開始する。ロクロニウム臭化物0.6～1.0mg/kg にて筋弛緩効果を得た後、酸素および3～5％セボフルランにてマスク換気を継続した後、気管挿管を行う。なお、入眠直後にBioScan 920-Ⅱ system の電極を貼付し、体内成分値の測定を行う。

・血液検査項目：ケトン体、遊離脂肪酸、血糖、インスリン、rapid turnover protein（RTP；レチノール結合蛋白）、レプチン、アディポネクチン、3-メチルヒスチジン

　乳幼児では、ケトン体、遊離脂肪酸、血糖、インスリン、レチノール結合蛋白のみ

・Bioelectrical Impedance Analysis（BIA）法（BioScan 920-Ⅱ system）による体内成分の測定項目：安静時代謝率、除脂肪量、体細胞量、除脂肪率、体脂肪率、体内総水分量、体内総水分率、細胞外水分量、細胞内水分量、細胞外水分率、細胞内水分率、細胞外質量、蛋白質量、ミネラル量、筋肉量、体内総カリウム量、体内総カルシウム量、グリコーゲン量、ドライウエイト、細胞外固形物、細胞外液、血漿量、血管外細胞間液、体容積、体密度

＊rapid turnover protein（RTP）は、血中半減期が短く、動的栄養状態指標の中でも測　定精度が高く、安定した結果が得られるため、栄養状態の改善や悪化を早期に知る指標とされている。特に、レチノール結合蛋白は、血中半減期0.5日と最も短い。

＊レプチンは脂肪細胞が出すホルモンで、視床下部に作用して満腹感を感じさせ、エネルギー代謝を制御し、体重増加を抑制する作用を示す。また、経日的なレプチンの低下と新生児の成長が相関すると報告されている（Wang LJ, et al: Kaohsiung J Med Sci. 10: 521, 2012）。

＊アディポネクチンは、インスリン受容体を介さない糖取り込み促進作用、[脂肪酸](https://ja.wikipedia.org/wiki/脂肪酸)の燃焼、細胞内の脂肪酸を減少してインスリン受容体の感受性を上げる作用、肝臓の[AMP](https://ja.wikipedia.org/wiki/AMP)キナーゼを活性化させることによる[インスリン](https://ja.wikipedia.org/wiki/インスリン)感受性の亢進、[動脈硬化](https://ja.wikipedia.org/wiki/動脈硬化)抑制、抗炎症、[心筋肥大](https://ja.wikipedia.org/w/index.php?title=心筋肥大&action=edit&redlink=1)抑制などの作用が報告されている。

＊3-メチルヒスチジンは、栄養状態の改善を判断する指標のひとつで、筋線維タンパクである アクチンとミオシンの構成アミノ酸である 。重度侵襲下や飢餓状態では、筋タンパクの異化が亢進し、筋線維タンパクの分解により遊離した3-メチルヒスチジンの血中および尿中濃度が上昇する。

７）その後、成人では麻酔開始2時間後までは非ブドウ糖含有液（生理食塩水等）による輸液を継続し、2時間を超えると1%ブドウ糖含有液（フィジオ140）に変更する（輸液速度：2～5mL/kg/h）。乳幼児・小児では、輸液を1%ブドウ糖含有製剤（フィジオ140）に変更し、ブドウ糖量にして120～200mg/kg/h となるように輸液を行う（Leelanukrom R, et al: Paediatr J Anesth 10: 353, 2000）。

８）通常の全身麻酔管理を行う。麻酔中に動脈血液ガス分析を1回行う。

９）麻酔導入2時間後を目安に2回目の採血（ケトン体、遊離脂肪酸、血糖、インスリン、レチノール結合蛋白、レプチン、アディポネクチン、3-メチルヒスチジン：採血量5.0cc）を行う。同時に、体内成分値測定を行う。これを行えない場合は、動脈血液ガス分析のために採血した血液の一部を検査用に使用する。なお、乳幼児・小児に対しては、採血量を軽減する目的で、1回目および2回目の採血でケトン体、遊離脂肪酸、血糖、インスリン、レチノール結合蛋白（採血量3.0cc）のみを測定する。

10）得られた検体を検査会社へ外注し、測定を行う。

（2）評価項目

Primary：成人において、術前のブドウ糖投与の有無により、血中ケトン体、遊離脂肪酸、血糖、インスリン、レチノール結合蛋白、レプチン量、アディポネクチン、3-メチルヒスチジンおよびBioelectrical Impedance Analysis（BIA）法にて測定した体内成分値を比較する。

Secondary：上記の各項目を乳幼児・小児で分析する。

（3）予定症例数

　　　成人において、過去に術前のブドウ糖投与による糖脂質代謝を検討した報告はないので、術中にブドウ糖を含有した輸液と含有しない輸液を投与した群間の比較データから、α=0.05、β=0.2として必要なサンプルサイズを計算すると28例（各群14例）となり、20%が脱落すると計算すると、成人で必要な症例数は34例（各群17例）である。乳幼児、小児については、ブドウ糖を含有する飲料を飲用することから必要症例数は算出できないが、成人と同様に17例とする。したがって、研究全体で必要な症例数は51例である。

（4）倫理的事項：

本研究では、全て通常の全身麻酔管理を行い、追加的に患者に対して侵襲を加えることはないため、安全性は通常の全身麻酔と同様であり、倫理的にも問題はない。Bioelectrical Impedance Analysis（BIA）法による体内成分値測定に関しては、心電図と同様のシール電極8枚を貼付し、インピーダンス法にて測定するので、生体に対して全く侵襲のない検査法であるので、倫理的な問題はない。また、術中に合計10.0 cc（乳幼児・小児では6.0cc）の採血を行うことも、生体には全く影響のない少ない量である。

5. スケジュールあるいは研究期間

（1）スケジュール

　　　　　　 　 　　病　　棟　　　　　　　　 　　　　手　術　室

時間経過

　　　　　　　　　　　入院当日　　　　 手術当日

全身麻酔関連 　　 （術前回診）　　　　術前絶飲食　　　　　　　全身麻酔

研究関連　　　　　　BIA法による　　　　　　　　　　　採血2回（成人各5.0ccずつ、

体内成分値測定　　　　　　　　　　　　　　　　　　乳幼児各3.0ccずつ）

　　　　　　　　　　　　　　　　　　　　　　　　　　　BIA法による体内成分値測定2回

＊ボディコンポジションアナライザーBioScan 920-Ⅱ systemは、全身電気的インピーダンスの技術を用いて、身体組成と体液の変動値を非侵襲的に測定する装置である。生きている有機体の細胞間と細胞外には体液があり、その体液は伝導体として働く。また、細胞膜は電気的にコンデンサーとして働き、不完全なリアクタンス素子とみなされる。生体電気インピーダンスの原理はすでに確立されているが、本装置は、生体内の電流条件に基づいて、身体インピーダンス、位相角、抵抗値、リアクタンスを決定する。これらの測定により、安静時代謝率、体細胞量、体脂肪率、体内総水分量、細胞外水分量、細胞内水分量、蛋白質量、筋肉量、グリコーゲン量、血漿量などの体内成分値の評価が可能になった。したがって、本研究にBioScan 920-Ⅱ systemを用いることは、被験者の絶飲絶食による体内成分値の変化を的確にとらえ、糖脂質代謝の変化を評価する上で有用な情報を提供するものと考えられる。

　　（2）期間：研究倫理審査委員会承認日　～　平成30年3月31日

　５．研究対象者の選定方針

適格基準：

　　（１）全身麻酔下で歯科・口腔外科手術を受ける被験者のうち、糖脂質代謝に影響を与える疾患（糖尿病など）を有さない被験者（ASA PS値1または2）

　　（２）本研究計画について十分に理解し、同意が可能な被験者。16歳未満の者については、代諾者による同意が可能な被験者

　　除外基準：

　　（１）コントロール不良の全身的合併症を有する被験者（ASA PS値3以上）

　　（２）糖脂質代謝に影響を与える疾患（糖尿病など）を有する被験者

（３）その他研究者が被験者として適当でないと判断した被験者

　　中止基準：

　　（１）全身麻酔中に全身状態が悪化した場合

（２）何らかの理由で予定量の採血が困難であった場合

（３）同意の撤回があった場合

　６．研究の科学的合理性の根拠

現在、世界中で広く普及している米国麻酔科学会および日本麻酔科学会の「術前の絶飲絶食ガイドライン」では、clear liquid は2 時間前まで許可されているが、これは血糖値の推移（低血糖を防止する）を基に作成されたものである。しかし、糖脂質代謝（ケトーシスを予防する）を考慮すると、術前に投与するclear liquidには、ブドウ糖を添加するほうがよい可能性が高いと考えられるが、この領域の研究は行われていない。また、各年齢層別での術前の絶飲絶食と糖脂質代謝に関する報告も少なく、一定の見解は得られていないのが実情である。

本研究では、通常行われている術前絶飲絶食および麻酔管理を行うので、被験者に対して特別な負担を強いるものではない。また、本研究の成果により、術前の絶飲絶食（ブドウ糖の投与の有無も含む）による糖脂質代謝の変化が明らかになり、将来「術前の絶飲絶食ガイドライン」の改善の可能性にも繋がるため、意義の大きい研究である。

　７．インフォームド・コンセントを受ける手続き

　1）**☑**新たに試料・情報を取得して研究を実施

本研究の対象者のうち16歳未満の被験者に対しては、代諾者からインフォームド・コンセントを得る。代諾者は原則として、親権者又は未成年後見人とする。研究実施責任者または研究分担者は、本研究に先立ち、被験者として適切と思われる者および代諾者に対し、本研究について、別添説明文書を用いて十分な説明をする。その際、質問する機会と研究に参加するか否かを判断するのに、十分な時間を与える。説明文書を用いた説明の後、本研究への参加についての判断は被験者本人または代諾者の自由意思による。参加の有無により被験者の診断や治療について利益又は不利益になるようなことはなく、また、一旦同意した後に、同意を取り消すことも可能であり、同意を取り消したことにより、被験者に不利益になることもない旨、被験者に十分説明した上で同意を取得する。被験者本人または代諾者の自由意思による同意が得られたときは、同意書に被験者の署名及び同意日を得る。説明を行った医師は、同意書に署名し、説明日を記入する。

被験者または代諾者が一旦同意した後に、同意を取り消す場合は、同意撤回書を渡し記載を行ってもらう。また、被験者が本研究に参加している期間に説明書または同意書が改定された場合は、その都度、同意の再取得を行う。

　　2）**□**自らの研究機関において保有している既存試料・情報を用いて研究・・別紙１参照

　　3）**□**他の研究機関に既存資料・情報を提供しようとする場合・・別紙１参照

　　4）**□**３の手続きに基づく既存試料・情報の提供を受けて研究を実施しようとする場合・別紙１参照

　８．個人情報等の取扱い

被験者から取得するデータには、氏名や診断名の個人情報が記録されるが、個人情報の取り扱いには十分注意し、情報の紛失や被験者個人が特定されることを防ぐため、以下に記載している措置を徹底する。

　　　採取した血液試料及び診療情報は、神奈川歯科大学大学院全身管理医歯学講座麻酔科学分野において、被験者氏名、生年月日、カルテ番号、住所、電話番号を消去し、代替する登録番号にて連結可能匿名化する。登録番号と被験者個人を連結する対応表は、同科内の外部と接続できないパソコンで管理し、対応表のファイルにはパスワードを設定する。このパソコンを設置する部屋の鍵は研究実施責任者のみが保有しており、入退室を管理する。個人情報管理責任者は、全身管理医歯学講座・教授・森本佳成である。

　　　なお、採血した検体は、上記連結可能匿名化を行ったうえで検査業者へ渡し測定を行う。

９．研究対象者に生じる負担並びに予測されるリスク及び利益、これらの総合的評価並びに当該負担及びリスクを最小化する対策

　　(1)　研究対象者に生じる負担：被験者から合計10.0cc（乳幼児では6.0cc）の研究用採血を行う。

　　(2)　予測されるリスク及び利益：本研究により被験者が直接受けることができる利益はない。

通常よりも採血の量がわずかに増えるが、健康上問題ない量であると考えられる。また、採血は静脈路確保時や全身麻酔中に行うので、被験者のストレスもない。

　　(3)　総合的評価：被験者が受けるリスクは少ない。

　　(4)　当該負担及びリスクを最小化する対策：採血は、静脈路確保時や術中に行うので、被験者のストレスはない。また、術中に通常の血液検査を行う場合は、その時に合わせて採血を行う。万が一、採血時に問題が発生した被験者についてはすぐに研究を中止し、担当医師が適切な対応を取る。

１０．試料・情報(研究に用いられる情報に係る資料を含む)の保管および廃棄の方法

　　1)保管方法：本研究において採取した試料、得られたデータ等は、神奈川歯科大学大学院全身管理医歯学講座麻酔科学分野において、同講座教授　森本佳成　責任の下、研究期間終了後5年間保存する。

　　2)廃棄方法：登録番号等を消去し、医療廃棄物として廃棄する。試料、データ等を将来別の研究に二次利用する予定はない。

１１．研究機関の長への報告内容及び方法

　　1年に1回の研究進捗状況報告および研究終了時に研究終了報告を行う。

１２．研究の資金源等、研究機関の研究に係わる利益相反及び個人の収益等、研究者等の研究に係わる

利益相反に関する状況

　　1)資金源

　　　①**□** 講座(分野)研究費　　　**☑** 科学研究費補助金(種目：基盤研究（C）)

**□** その他(　　　　　 　)

　　2)利益相反（COI）

**□**利益相反（COI）委員会申請中

**□**利益相反（COI）委員会承認済み

**☑**該当なし

１３．研究に関する情報公開の方法

本研究の成果は国際・国内学会発表及び論文発表を予定している。その際、個人を識別できる情報は一切含まない。

１４．研究対象者等及びその関係者からの相談等への対応

　　　本研究に関する質問や問い合わせは、実施責任者である全身管理医歯学講座・教授・森本佳成　　まで連絡いただく。

１５．代諾者等からインフォームド・コンセントを受ける場合の手続き

　　１）**□**該当しない

　　２）**☑**該当する（手続き：被験者が16歳未満または研究参加の可否を自身で判断できない場合は、被験者および代諾者に対し、説明文書をもって説明し、同意文書にて同意を得る。）

１６．インフォームド・アセントを得る場合の手続き

　　１）**□**該当しない

　　２）**☑**該当する（手続き：被験者が16歳未満または研究参加の可否を自身で判断できない場合は、被験者および代諾者に対し、説明文書をもって説明し、同意文書にて同意を得る。）

１７．研究対象者に緊急かつ明白な生命の危機が生じている状況における研究を実施する場合

　　倫理指針第12の５の規定に掲げる要件のすべてを満たしていることについて判断する方法

**□**該当　判断する方法：

**☑**該当しない

１８．研究対象者等に経済的負担又は謝礼がある場合、その旨及びその内容

１）費用負担　**☑**無　（ただし、通常の手術や麻酔等の診療費用はご負担いただきます。）

**□**有　(内容：　　　　　　　　　　　　　　　　　　　　　　)

２）謝 礼　**☑**無

**□**有　(内容：　　　　　　　　　　　　　　　　　　　　　　)

１９．侵襲(軽微な侵襲を除く)を伴う研究の場合、その旨及びその内容

　　１）**☑**該当しない

　　２）**□**該当する（内容：　　　　　　）

２０．侵襲を伴う研究の場合、当該研究によって生じた健康被害に対する補償の有無及びその内容

　　１）**☑**該当しない

　　２）**□**該当する（内容：　　　　　　）

２１．通常の診療を超える医療行為を伴う研究の場合、研究対象者への研究実施後における医療の

提供に関する対応

　　１）**☑**該当しない

　　２）**□**該当する（対応：　　　　　）

２２．研究の実施に伴い、研究対象者の健康、子孫に受け継がれ得る遺伝的特徴等に関する重要な知見

が得られる可能性がある場合、研究対象者に係る研究結果(偶発的所見を含む)の取扱い

　　１）**☑**該当しない

　　２）**□**該当する（取扱：　　　　　）

２３．研究に関する業務の一部を委託する場合、当該業務内容及び委託先の監督方法

　　１）**□**該当しない

　　２）**☑**該当する（検査会社に、採血した検体の測定を外注する。採取した血液試料は、神奈川歯科大学大学院全身管理医歯学講座麻酔科学分野において連結可能匿名化を行ったうえで、検査会社へ渡し測定を行う。）

２４．研究対象者から取得された試料・情報について、研究対象者等から同意を受ける時点では特定さ

れない将来の研究のために用いられる可能性又は他の研究機関に提供する可能性がある場合、その

旨と同意を受ける時点において想定される内容

　　１）**☑**該当しない

　　２）**□**該当する（内容：　　　　）

２５．倫理指針第20の規定によるモニタリング及び監査を実施する場合、その実施体制及び実施手順

　　１）**☑**該当しない

　　２）**□**該当する（　　　　　　　）

(様式3)

**研究説明書**

　１．研究の名称：周術期における糖脂質代謝の改善に関する研究―術前の絶飲絶食が糖脂質代謝に　及ぼす影響について

　　　この研究は、研究機関の長の許可を受けています。

　２．研究機関の名称：神奈川歯科大学大学院歯学研究科全身管理医歯学講座

　　　研究実施責任者：森本佳成　　　　　　　　連絡先：046-822-8842

　３．研究の目的及び意義

　（1）目的：全身麻酔にあたっては、麻酔中の嘔吐や誤嚥を防止するために、術前に数時間の絶飲絶食を行います。このとき、通常の術前飲水制限を行うと、糖分の量が不足して飢餓時に出現する血中ケトン体が大幅に増加する可能性があることが分かってきました。

通常、食事などからとれる糖分などが体の主なエネルギー源となります。しかし、長時間の絶飲絶食や激しい運動後では、糖分の不足により、体内に貯蔵された成分が分解されてケトン体が産生されます。この状態が持続すると、ケトン体は過度に増加し代謝異常をきたし、それによる病気を生じる危険性があります。しかし、全身麻酔前の絶飲絶食によるケトン体の上昇については過去に研究はなく、解明されていない問題です。また、ケトン体の発生を抑制する方法についても、まったく検討されていないのが現状です。

本研究の目的は、乳児・小児から成人までの各年齢層の患者さんの手術に際し、通常の術前絶飲絶食を行い、インピーダンス法（体内を通る微小な電流の抵抗を測定する方法）を用いて、患者さんの体内成分（体水分量、蛋白量など）の変化を測定するとともに、血液中の代謝の指標となる項目（ケトン体、血糖など）を測定し、現在広く行われている術前絶飲絶食が患者さんの代謝へ与える問題点を解明し、全身麻酔時の絶飲絶食の方法を見直すことをめざすことです。

（2）意義：本研究の結果、術前の絶飲絶食による代謝の異常が解明されます。これにより、これら代謝異常を是正するための的確な治療法（術前飲水の内容や術中輸液の内容の改善）を開発する手掛かりを見出すことができると考えられます。

　４．研究の方法及び期間

　（1）方法：

この研究では、神奈川歯科大学附属病院にて歯科・口腔外科手術を受けられる患者さんで、糖尿病などの糖脂質代謝に影響を与える病気をお持ちでない方を対象とさせていただく予定です。一方、糖尿病などの糖脂質代謝に影響を与えるご病気をお持ちの患者さんは、本研究にはご参加いただけません。また、この研究は観察研究とさせていただき、特別な介入（通常では行わない手技や薬剤投与等）は行いません。すなわち、通常の麻酔管理を行いますので、特別な負担は生じません。

この研究への参加に同意いただきますと、インピーダンス法による体内成分値の測定（スポーツジムなどにある体脂肪率などを測定する器械と同様の方法です）のほか、全身麻酔中に研究用の血液5.0ccずつ2回採血させていただきます（乳幼児では3.0ccずつ2回）。この研究用の血液に含まれるケトン体、血糖、インスリン、レチノール結合蛋白、レプチン、アディポネクチン、3-メチルヒスチジンの量を測定し、患者さんの体内成分値と併せて分析を行うことにより、手術時の糖脂質代謝の異常を調べます。

具体的な方法としては、

１）全身麻酔前日の夕方（16 時〜22 時）に、日常の体内成分値として、患者さんにインピーダンス法の電極を貼付し、体内成分の測定を行います。この装置は、両側手背および足背に心電図と同様のシール電極を8枚（各部位2枚×4ヶ所）を貼り、インピーダンス法にて測定しますので、生体に対しては全く侵襲なく、何ら影響を与えません。（10 秒程度で測定可能）。

＊BioScan 920-Ⅱ System（ボディコンポジションアナライザー）は、電極8枚を手足に貼り、この電極を通じて体内を流れる低レベルの信号を検知し、腕・体幹・脚を流れる身体全体のインピーダンスを測定します。このインピーダンスの測定により、体内の安静時代謝率、体細胞量、体脂肪率、体内総水分量、細胞外水分量、細胞内水分量、蛋白質量、筋肉量、グリコーゲン量、血漿量などの体内成分を測定します。この装置を用いることにより、絶飲摂食による体内成分値の変化を的確にとらえ、糖脂質代謝の変化を評価する上で有用な情報が得られると考えます。


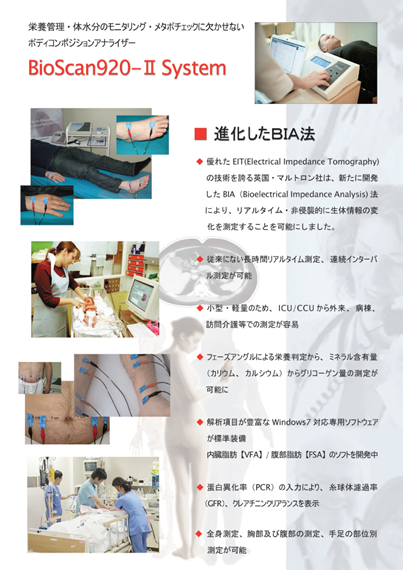


２）手術当日、一般的な術前の絶飲絶食（人工乳は麻酔5 時間前まで、普通の飲料は2 時間前まで許可）を行います。

①　乳幼児では、飲水量は目盛付きカップまたは哺乳瓶にて測定します。なお、母乳しか飲めない患児は本研究に含めません。

小児では、普通の飲料としてブドウ糖を含有する飲料を使用します。手術前日の夜からは、これしか飲むことはできません。

②　成人に対しては、普通の飲料として、ブドウ糖を含有しない飲料と含有する飲料のどちらかを指定します。手術前日の夜からは、これしか飲むことはできません。

３）成人では、手術室入室後、通常通り静脈路を確保し、この時に1回目の採血（採血量5.0cc）を行います。同時にインピーダンス法の電極を貼付し、体内成分値の測定を行います。その後、通法通りに全身麻酔を開始します。

４）乳幼児・小児では、先にガスの麻酔薬にて入眠してもらい、通常通り静脈路を確保し、この時に1回目の採血（採血量3.0cc）を行います。同時にインピーダンス法の電極を貼付し、体内成分値の測定を行います。その後、通法通りに全身麻酔を行います。

５）その後、非ブドウ糖含有液（生理食塩水等）またはブドウ糖を含む製剤にて輸液をします。

６）通常の麻酔管理を行います。通常通り、麻酔中に動脈血を採血し、呼吸状態や代謝の状態を確認します。

７）麻酔開始2時間後を目安に2回目の採血（5.0cc、乳幼児では3.0cc）を行います。同時に、2回目の体内成分値測定を行います。

８）得られたデータを、成人ではブドウ糖を含有しない飲料と含有する飲料の比較を行います。さらに、得られたデータを全年齢別に比較します。

＊測定項目

・血液検査項目は、ケトン体、遊離脂肪酸、血糖、インスリン、レチノール結合蛋白、レプチン、アディポネクチン、3-メチルヒスチジン

・インピーダンス法（BioScan 920-Ⅱ system）による体内成分値の測定

　　　　　　 　 　　病　　棟　　　　　　　　 　　　　手　術　室

時間経過

　　　　　　　　　　　入院当日　　　　 手術当日

全身麻酔関連 　　 （術前回診）　　　　術前絶飲食　　　　　　　全身麻酔

研究関連　　　　　　BIA法による　　　　　　　　　　　採血2回（成人各5.0ccずつ、

体内成分値測定　　　　　　　　　　　　　　　　　　乳幼児各3.0ccずつ）

　　　　　　　　　　　　　　　　　　　　　　　　　　　BIA法による体内成分値測定2回

（2）期間：研究倫理審査委員会承認日～平成30年3月31日

５．研究対象者として選定された理由

全身麻酔下で歯科・口腔外科手術を受ける患者さんのうち、糖脂質代謝に影響を与える疾患（糖尿病など）や、コントロール不良の全身的合併症を有さない患者さんは、本研究の対象者と選定されています。

６．研究対象者に生じる負担並びに予測されるリスク及び利益

　　(1)　研究対象者に生じる負担：研究対象者から合計10.0cc（乳幼児では6.0cc）の研究用採血を行います。通常よりも採血の量がわずかに増えますが、健康上問題ない量であると考えられます。また、採血は静脈路確保時または全身麻酔中に行いますので、研究対象者のストレスもありません。

　　(2)　予測されるリスク及び利益：本研究により研究対象者が直接受けることができる利益はありません。

通常よりも採血の量がわずかに増えますが、健康上問題ない量であると考えられます。また、採血は静脈路確保時または全身麻酔中に行いますので、研究対象者のストレスもありません。万が一、採血時に問題が発生した研究対象者については、すぐに研究を中止し、担当医師が適切な対応を取ります。

７．研究が実施又は継続されることに同意した場合であっても随時これを撤回できること

８．研究が実施又は継続されることに同意しないこと又は同意を撤回することによって研究対象者等

が不利益な扱いを受けないこと

　　　この研究への参加は研究対象者ご本人または代諾者の自由な意思で決めてください。同意されなくても、患者さんの診断や治療に不利益になることは全くありません。また、いったん同意した場合でも、ご本人または代諾者が研究の中止を希望された場合は、患者さんが不利益を受けることなく、いつでも同意を取り消すことができます。その場合は、研究用に採取した血液やその血液を調べた結果などは廃棄され、カルテの情報もそれ以降はこの研究目的に用いられることはありません。ただし、同意を取り消した時にすでに研究結果が論文などで公表されていた場合や、データや検体等が完全に匿名化されて特定できない場合には、完全に廃棄できないことがあります。

採血の際に研究のために採らせていただく血液の量も健康上問題のない量と考えておりますが、もし、採血の際に問題が発生した場合は、すぐに研究を中止し、研究担当医師が誠意をもって対応いたします。また、全身麻酔や手術等による問題が発生した場合も、研究は中止します。

９．研究に関する情報公開の方法

　本研究成果は、国内または国外の学会にて発表し、論文として公表する予定です。

１０．研究に関する資料の開示の方法

　　この研究に関して、参加の継続についてご本人の意思に影響を与える可能性がある情報が得られた場合には、すみやかにお伝えします。また、ご本人や代諾者の方のご希望により、この研究に参加してくださった方々の個人情報の保護や、この研究の独創性の確保に支障がない範囲で、この研究の計画書や研究の方法に関する資料をご覧いただくことができます。資料の閲覧を希望される方は、どうぞお申し出ください。

１１．個人情報等の取扱い

患者さんの体内成分値や血液を測定した結果やカルテに含まれる情報をこの研究に使用する際には、患者さんのお名前の代わりに研究用の番号を付けて取り扱います。患者さんと研究用の番号を結びつける対応表のファイルにはパスワードを設定し、神奈川歯科大学大学院歯学研究科全身管理医歯学講座麻酔科学分野内のインターネットに接続できないパソコンに保存します。このパソコンが設置されている部屋は、同科職員によって入室が管理されており、第三者が立ち入ることはできません。

　　また、この研究の成果を発表したり、それを元に特許等の申請をしたりする場合にも、あなたが特定できる情報を使用することはありません。

この研究によって取得した個人情報は、神奈川歯科大学大学院歯学研究科全身管理医歯学講座・教授・森本佳成の責任の下、厳重な管理を行います。

１２．試料・情報の保管及び廃棄の方法

　１）保管方法：本研究において採取した試料、得られたデータ等は、神奈川歯科大学大学院全身管理医歯学講座麻酔科学分野において、同講座教授　森本佳成　責任の下、研究期間終了後5年間保存します。

　２）廃棄の方法：登録番号等を消去し、医療廃棄物として廃棄します。試料、データ等を将来別の研究に二次利用する予定はありません。

１３．研究の資金源等、研究機関の研究に係わる利益相反及び個人の収益等、研究者等の研究に係る

利益相反に関する状況

この研究に関して必要な費用は、文部科学省科学研究費補助金等による研究費でまかなわれますので、あなたに通常の治療費以外に新たな負担を求めることはありません。また、あなたに謝礼をお渡しすることもありません。

1)資金源

　　　①**□** 講座(分野)研究費　**☑** 科学研究費補助金(種目：基盤研究（C）)　**□** その他(　　 　)

　　2)利益相反（COI）

**□**利益相反（COI）委員会申請中

**□**利益相反（COI）委員会承認済み

**☑**該当なし

１４．研究対象者等及びその関係者からの相談等への対応について

研究対象者及び代諾者の方で、本研究に関する相談のご希望があれば、神奈川歯科大学大学院全身管理医歯学講座麻酔科学分野の担当者までお申し出ください。実施責任者または分担研究者がご相談に対応させていただきます。

１５．研究対象者等に経済的負担又は謝礼について

１）費用負担　**☑**無　（ただし、通常の手術や麻酔等の診療費用はご負担いただきます。）

**□**有　(内容：　　　　　　　　　　　　　　　　　　　　　　)

２）謝 礼　**☑**無

**□**有　(内容：　　　　　　　　　　　　　　　　　　　　　　)

１６．通常の診療を超える医療行為を伴う研究について

　　□他の治療法等に関する事項

１）**☑**該当しない

　　　２）**□**該当する（事項：　　　　　　　　　　　　　）

　　□研究対象者への研究実施後における医療の提供に関する対応

１）**☑**該当しない

　　　２）**□**該当する（対応：　　　　　　　　　　　　　　　　　　　　　　　）

１７．研究の実施に伴い、研究対象者の健康、子孫に受け継がれ得る遺伝的特徴等に関する重要な知見

が得られる可能性がある場合について

１）**☑**該当しない

　　２）**□**該当する（対応：　　　　　　　　　　　　　　　　　　　　　　　　）

１８．侵襲を伴う研究について

１）**☑**該当しない

　　２）**□**該当する（内容：　　　　　　　　　　　　　　　　　　　　　　　　）

１９．取得された試料・情報について、将来の研究等で用いられる場合について

　１）**☑**該当しない

２）**□**該当する（内容：　　　　　　　　　　　　　　　　　　　　　　　　　　　）

２０．侵襲（軽微な侵襲を除く。）を伴う研究であって介入を行う場合について

１）**☑**該当しない

　　２）**□**該当する（対応：　　　　　　　　　　　　　　　　　　　　　　　　　　　）
