# Supplementary material for: Effects of preoperative oral carbohydrate intake on catabolism, nutrition and adipocytokines during minor surgery: A randomized, prospective, controlled clinical phase II trial
Source: PLoS One. 2019 May 13;14(5):e0216525. doi: 10.1371/journal.pone.0216525 (PMC6513065; doi:10.1371/journal.pone.0216525)
Supplement: S3 Fig — (DOC) [file pone.0216525.s003.doc]

**
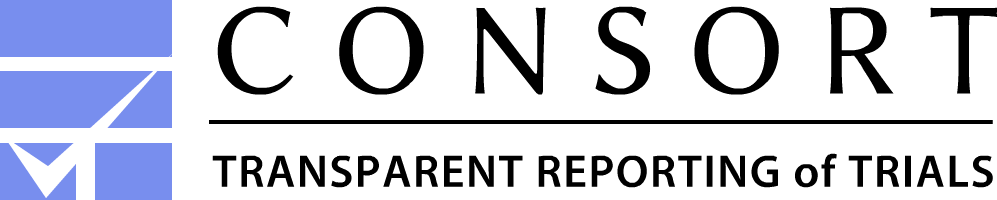
**

**CONSORT 2010 Flow Diagram**

**Allocation**

**Analysis**

**Follow-Up**

**Enrollment**

Assessed for eligibility (n=43)

Excluded (n=9)

♦  Not meeting inclusion criteria (n=0)

♦  Declined to participate (n=9)

♦  Other reasons (n=0)

Analysed (n=16)
♦ Excluded from analysis (give reasons) (n=0)

Lost to follow-up (decline to drink Arginaid Water®) (n=1) Discontinued intervention (give reasons) (n=0)

Allocated to intervention (n=17)

♦ Received allocated intervention (n=17)

♦ Did not receive allocated intervention (give reasons) (n=0)

Lost to follow-up (Length of operation<2hr) (n=1) Discontinued intervention (give reasons) (n=0)

Allocated to intervention (n=17)

♦ Received allocated intervention (n=17)

♦ Did not receive allocated intervention (give reasons) (n=0)

Analysed (n=16)
♦ Excluded from analysis (give reasons) (n=0)

Randomized (n=34)
